# Seasonal Variation in Public Interest in Gout: Longitudinal Infodemiology Study Using Google Trends (2014–2024)

**DOI:** 10.2196/75415

**Published:** 2025-08-27

**Authors:** Naomi Schlesinger, Ioannis P Androulakis

**Affiliations:** 1 Division of Rheumatology, Department of Medicine, Spencer Fox Eccles School of Medicine, University of Utah, Salt Lake City, UT, United States; 2 Biomedical Engineering, Rutgers, The State University of New Jersey, 599 Taylor Rd, Piscataway, NJ, 08854, United States, 1 8484456561

**Keywords:** gout, Google Trends, seasonal variation, infodemiology, search behavior, public health, seasonality

## Abstract

**Background:**

Gout, the most common inflammatory arthritis worldwide, shows clear seasonal variation in flares. Traditional epidemiology provides important insights but often lacks real-time resolution. Digital behavior, such as online search patterns, offers a scalable, timely complement that can capture seasonal trends in disease-related activity.

**Objective:**

This study aimed to determine whether public interest in gout, as expressed through Google (Google LLC) search queries, exhibits seasonal variation across countries, US states, and metropolitan areas, and to assess the influence of symptom- and language-specific search terms. We evaluated whether a bimodal (semiannual) seasonal pattern better described certain queries, providing further insight into complex behavioral rhythms.

**Methods:**

We retrieved monthly Google Trends data for gout-related queries from January 1, 2014, to December 31, 2024, covering 70 countries, all 50 US states, and 36 major cities in the United States and Canada. Queries included generic terms, symptom descriptors, and language-specific translations in 14 languages. We applied cosinor modeling to assess seasonality and calculated the amplitude and phase of fitted sinusoidal curves. Significance was assessed using *P* values and adjusted for multiple comparisons using the Benjamini–Hochberg false discovery rate (FDR) within and across subgroups, and the Bonferroni method. To explore bimodal patterns, we compared 12- and 6-month harmonics using changes in Akaike and Bayesian information criteria.

**Results:**

We found robust seasonal variation in gout-related search interest across multiple geographic and linguistic categories. Statistically significant seasonality (original *P*<.05) was detected in 41 of 70 countries using English terms, with 36 remaining significant after within-group FDR correction and 39 under pooled FDR; only 7 remained significant after Bonferroni adjustment. Among 20 countries using language-specific queries, 15 showed consistent seasonality across all 3 non-Bonferroni methods, while 2 met the Bonferroni threshold. In the United States, 49 out of 50 states and 33 out of 36 cities demonstrated significant seasonality (Bonferroni-adjusted significance in 9 and 10 units, respectively). For symptom- and treatment-related search terms, 18 of 21 exhibited seasonality under multiple correction methods. Peaks in search volume generally occurred in late spring or early summer in the northern hemisphere, with corresponding seasonal shifts in the southern hemisphere. Bimodal patterns were uncommon but identified for terms such as “obesity” and “swollen big toe,” suggesting more complex cyclic interest in certain contexts.

**Conclusions:**

Google search activity reflects the seasonal dynamics of gout flares, highlighting infodemiology as a population-scale complement to traditional surveillance. This approach may anticipate care needs, guide digital health strategies, and improve preparedness for seasonal, climate-sensitive conditions, while emphasizing the importance of geographic, climatic, and linguistic context in interpreting trends.

## Introduction

Gout is the most prevalent inflammatory arthritis globally, recently identified as an auto-inflammatory disease [1]. Gout is a metabolic disorder characterized by an elevated uric acid pool leading to the formation and deposition of monosodium urate (MSU) crystals. The prevalence of gout is on the rise, partly due to the obesity epidemic, impacting 5.1% (12.1 million) of US adults, 5.9% of men, and 2.0% of women [1,2]. The likelihood of developing gout increases with age, affecting 10% of non-Hispanic Whites aged ≥65 years and 14.8% of Asians aged ≥65 years, although it can affect adults of any age [3,4]. The primary goals of gout treatment are to prevent the formation of MSU crystals, reduce their deposition, and reduce inflammation. Gout flares exhibit a strong seasonal pattern, with incidence peaking in the spring or summer [5-9], leading to a seasonal trend related to serum urate levels fluctuating seasonally, which are highest in the summer. Furthermore, the functioning of the immune system in vertebrates shows significant seasonal variation, with heightened immune responses and increased disease severity during the shorter winter days as organisms adjust to the cold-related stresses of winter [10]. Research indicates that levels of interleukin-6, its soluble receptor, and C-reactive protein (CRP) are elevated during the summer and winter months [11,12]. In addition, the expression of proinflammatory messenger ribonucleic acid (mRNA) and proteins in peripheral blood mononuclear cells is more pronounced during the winter [13]. These inflammatory markers are risk indicators for inflammatory diseases, which show seasonal patterns of increased prevalence and worsening symptoms during the winter and spring [14]. This adaptation is critical for maintaining immune competence, while failure to adapt could impair the immune system’s effectiveness. The microbiome’s critical role was recently identified as a likely contributor to gout’s seasonal character [15]. Since distinct, cross-regulating seasonal activity patterns characterize both metabolic and immune mediators, it is critical to better describe these interacting dynamics, which shift the balance of power and tip the scale, initiating an inflammatory reaction and an episode. Neglecting this dynamic balance hampers our understanding and limits the clinical utility of gout’s seasonal patterns.

Traditional methods for tracking the seasonality of chronic inflammatory diseases involve epidemiological approaches that gather and analyze clinical and environmental data and population health metrics over time. These methods are fundamental to understanding patterns and trends that can influence disease management and policy and include: (1) clinical studies [16] and longitudinal trials involving structured and controlled setups where data on symptom onset, progression, and season-related flare-ups are collected; (2) health records analysis [17,18] examining patient records from health care providers can offer insights into the timing and frequency of disease flare-ups. This method relies on the availability and accuracy of health care data and can identify trends in symptom management needs throughout the year. In Åkerblom et al [19], the analysis of 170,915 urate test results from patients at a Swedish tertiary care hospital between 2000 and 2016 revealed concentration peaks in the summer months. In Kurajoh et al [20], a large-scale database of medical claims in Japan filed between January 2019 and December 2022 was accessed, revealing a statistically significantly higher rate of prescriptions of urate-lowering drugs; (3) population-based health databases [11] using large-scale health data to help infer disease seasonality at the population level. By overlaying health data with climatic or environmental information, researchers can more precisely identify and predict seasonal patterns in disease activity.

These methods provide foundational data that help understand disease mechanisms and the impact of seasonal changes on disease dynamics. However, they often require considerable time and resources, may not provide real-time data, and can be slow and costly, limiting the ability to rapidly respond to emerging health challenges.

In contrast to traditional epidemiological methods, digital data sources such as search engines and social media platforms offer immediate, scalable insights into public perceptions and behaviors. By analyzing digital footprints, researchers can rapidly detect shifts in health concerns and respond more dynamically to seasonal patterns in disease activity. This capability enables clinicians and public health professionals to anticipate flare-ups and allocate resources more effectively. With near-universal internet access, individuals are increasingly turning to the web for health-related information, seeking answers about symptoms, causes, and treatments. This shift in behavior is exemplified by the well-documented “Dr. Google” phenomenon, where the internet serves as a first stop for health inquiry, often preceding formal medical consultation [21].

Infodemiology is a term coined by health informatics specialist Gunther Eysenbach [22]. It refers to studying the distribution and determinants of information on the internet and its impact on public health and policy. As a discipline, infodemiology is particularly valuable in tracking and analyzing the dynamics of chronic inflammatory diseases, especially in understanding their seasonality, how symptoms and disease activity may fluctuate with changes in the environment across different times of the year. By using vast amounts of data generated from digital platforms, infodemiology offers insights into public health trends that are not easily captured through traditional epidemiological methods. For instance, search engine queries can reveal the frequency and pattern of searches related to symptoms and treatments of chronic inflammatory diseases. Analyzing these search trends over time allows researchers to identify when interest or concern about specific symptoms peaks, which might indicate seasonal exacerbations. Such data can highlight when patients are experiencing more severe symptoms, prompting them to seek online information. For example, in [23] Google queries related to gout were analyzed, revealing a statistically significant seasonal variation in relative volume, whereas in [24], Google searches were correlated with health insurance data, establishing a likely gout seasonal variation. Integrating environmental data with infodemiology enhances the study of disease seasonality by linking online activity, such as spikes in search queries or social media discussions, to external factors like weather changes. This approach helps clarify causal relationships between environmental triggers and symptom fluctuations, improving our ability to forecast trends.

In this study, we investigate whether public interest in gout, as reflected in Google search behavior, displays meaningful seasonal variation and whether this digital behavior aligns with the clinical seasonality of gout flares. By examining Google Trends (Google LLC) data from a global sample of 70 countries, 50 US states, and 36 major metropolitan areas in North America, along with 20 symptom- and treatment-related search terms and language-specific variants, we aim to determine the presence, amplitude, and timing of seasonal patterns in information-seeking behavior. In doing so, we assess the utility of infodemiology as a complementary tool for studying disease seasonality and generating insights to inform health communication, policy, and care delivery.

## Methods

### Google Trends

Google dominates the global search engine market, with a share consistently exceeding 80% and reaching over 90% in some regions [25]. This translates to a staggering volume of approximately 8.5 billion daily searches. While competitors like Bing (Microsoft Corporation; 3%), Baidu (Baidu, Inc; 1%), Yandex (Yandex NV; 1%), or Yahoo (Yahoo! Inc; 1%) exist, Google’s influence on how people find information online is undeniable and shapes the very way we interact with the web. Google Trends [26] is a rich, publicly accessible tool that provides insights into the relative popularity of Google search terms and topics over time [27]. Rather than offering raw search volumes, it provides relative Google search requests to reveal trends and patterns in search interest on a normalized scale. Rather than showing raw reporting counts, Google analyzes a sample of searches and normalizes the data on a scale of 0 to 100. A value of 100 represents the highest relative popularity for that term within the chosen timeframe and location, while a value of 50 indicates half the popularity. A value of 0 means there was not enough search data for that term. It is important to note that Google Trends data are normalized independently for each query, with values scaled from 0 to 100 based on relative search volume within the selected region and time period. As such, direct quantitative comparisons between groups are not appropriate. However, if the goal of the analysis is not to compare absolute search volumes across regions or groups, but rather to evaluate the presence and timing of seasonal patterns within each group independently, Google Trends is a suitable and informative tool.

Google Trends is invaluable for understanding how public attention and information-seeking behavior evolve in various domains, including health and disease awareness. In the context of gout, Google Trends offers a window into the temporal variations of user queries related to this condition, potentially reflecting fluctuations in symptom experience or public interest triggered by disease prevalence. Google Trends data on “gout” encompasses a historical record of how frequently and when users have included this term in their search queries across various geographic regions and time frames. The platform allows researchers to visualize this search interest over time, compare interest across different locations, and filter the results by categories and related topics that users might combine with “gout.” Since Google Trends provides data spanning the last 20 years, researchers interested in the seasonality of gout have the opportunity to examine long-term trends and potential recurring annual patterns. It is important to note that Google Trends data have limitations. It reflects search interest, which may correlate with but does not directly equal gout incidence or severity. External events like news articles or awareness campaigns might influence search behavior.

The longitudinal nature of Google Trends data and its geographical granularity and insights into related user queries make it a valuable starting point for generating hypotheses about gout seasonality. These hypotheses could then be further investigated with epidemiological studies or targeted surveys designed to pinpoint the precise drivers behind the observed seasonal trends in search interest. Google Trends data reflects the relative popularity of a search term over time within a specific geographic region.

Although Google Trends reports query information as of January 1, 2004, based on a preliminary review, we opted to concentrate on the last decade (January 1, 2014–December 31, 2024). This date was chosen for several reasons: Google Trends data become increasingly reliable and comprehensive in more recent years, ensuring that the analysis is based on a robust volume of search data and reducing the impact of potential anomalies or periods with lower search activity; using data from 2014 onwards minimizes the risk of inconsistencies or distortions that might arise from earlier changes in the platform’s data collection or normalization methodologies; focusing on this more recent period ensures that the analysis is relevant to current trends and patterns, capturing a period of significant growth in internet usage and the evolution of search behavior in the digital age; starting in 2014 allows for a reasonably long observation window, providing sufficient data points to identify meaningful trends and patterns, while avoiding the inclusion of data from a period where search behavior may have been fundamentally different; and finally, this timeframe minimizes the impact of earlier fluctuations in search query volume and user behavior on the internet, leading to more stable and reliable results.

To gain a comprehensive and nuanced understanding of the public’s seasonal interest in gout, we performed a series of structured queries across multiple geographies and keyword categories. First, we used the English term “gout” to assess global interest by analyzing query distributions in 70 countries, all 50 US states, and 36 major metropolitan areas in North America (including both the United States and Canada). The selection of countries included in this analysis was based on multiple criteria to ensure both global coverage and data quality. Second, we prioritized countries for which Google Trends provided sufficient and stable search volume data over the full study period, as low-volume regions often yield sparse or unreliable results. Thus, we required that Google Trends provide a recording for each month over the 10-year period. Third, the sample includes a broad geographic and socioeconomic representation, encompassing high-, middle-, and low-income countries across all inhabited continents. This diversity allows for the examination of seasonal patterns in relation to climate, culture, health care access, and digital engagement. Fourth, countries were included if they met minimum thresholds of internet penetration (25%) and population size likely to produce meaningful digital epidemiological signals (300,000). Finally, the inclusion of some smaller territories (eg, Guadeloupe and Martinique) reflects regions where gout is of known clinical or cultural relevance and where Google search data were available and interpretable. This multifactorial approach balances scientific rigor, geographic inclusivity, and data reliability**,** making the analysis generalizable while grounded in the constraints of infodemiologic research. Details about all countries can be found in Multimedia Appendix 1.

English remains the dominant language of medical and scientific communication globally, and many individuals—especially those with higher health literacy or access to international content—routinely search for health information in English, even in non-English-speaking countries. However, relying solely on English queries may obscure culturally specific patterns of information-seeking behavior, particularly among populations that engage more naturally with native-language search terms. Given the multilingual nature of global search behavior, we further expanded our analysis by incorporating 14 language-specific expressions for “gout” across 20 countries for the term “gout” in Czech (dna), Danish (urinsyregigt), Dutch (jicht), French (goutte), Finnish (kihti), German (gicht), Greek (ποδάγρα), Italian (gotta), Norwegian (urinsyregikt), Romanian (gută), Portuguese (gota), Spanish (gota), Hebrew (גאוט), and Swedish (gikt), we examined the query distribution in 20 countries around the globe. This subset was selected to ensure linguistic and geographic diversity across major language families and regions with high internet penetration and stable Google Trends query volume. These languages are spoken in countries with robust public health systems and widespread internet access, making them ideal for assessing how native-language search behavior might capture local interest in disease. In addition, several of the included countries, such as France, Germany, Israel, and Brazil, exhibit bilingual or multilingual internet search behavior, providing a rich basis for comparison between English and native search patterns. The inclusion of both Indo-European and Semitic languages further supports the generalizability of our findings and allows us to evaluate the degree to which linguistic framing influences seasonal interest patterns in health-related search behavior. Collectively, this targeted selection allows for the exploration of cultural and linguistic factors influencing public information-seeking behavior about gout, while maintaining analytic feasibility and consistency across countries, and finally to capture search behavior that may reflect early symptom exploration or postdiagnosis information-seeking, we analyzed searches for descriptive symptom terms (eg, “burning big toe” and “pain in the big toe”) as well as diagnosis- and treatment-oriented queries. We examined the frequency distribution of queries related to gout symptoms without mentioning the term gout reflecting public interest in symptoms whose cause is yet to be determined: “burning big toe,” “stiff big toe,” “big toe joint pain,” “pain in the big toe,” “sore big toe,” “throbbing toe pain,” and “swollen big toe”. Furthermore, we examined queries related to requesting information likely once a gout diagnosis has been provided or expressing the possibility of self-diagnosis: “gout doctor,” “gout diabetes,” “gout attack,” “gout flare,” “gout cure,” “gout diet,” “gout symptoms,” “gout high blood pressure,” “gout treatment,” “nonsteroidal anti-inflammatory drug (NSAID) gout,” and “prednisone gout.”

This multifaceted approach allowed us to better characterize both general and clinically-informed public interest in gout across cultural and linguistic contexts.

### Model Development and Analysis

The averaged monthly data are analyzed using a standard cosinor model, a harmonic regression tailored for analyzing and quantifying periodic (monthly in our case) variations observed in time-series data. This model fits a sinusoidal curve $y=M+Asin\left(\frac{2\pi t}{12}+\phi \right),$ to the monthly search frequency data, aiming to capture any inherent cyclic behavior over each year. Statistical significance for the amplitude parameter was assessed using a 2-tailed *t* test with a nominal α=0.05. We assessed significance using multiple comparisons, across all groups (countries using English search terms [n=70], countries using language-specific queries [n=20], US states [n=50], United States and Canada cities [n=36], symptoms [n=21]) rather than across all cases combined. To assess the significance of seasonality of search interest across multiple geographical units, we first applied cosinor regression to time-series Google Trends data collected over a 10-year period for each location. This yielded a *P* value for the statistical significance of the seasonal (periodic) component for each time series. We grouped these *P* values into 5 predefined subgroups based on geographic granularity: countries (n=70), US states (n=50), US cities (n=36), language specificity (n=20), and symptoms (n=21), resulting in a total of 197 individual tests. To account for multiple comparisons and evaluate the robustness of detected seasonal patterns, we applied 3 statistical correction procedures: (1) within-group false discovery rate (FDR**)** correction using the Benjamini–Hochberg procedure applied separately to each subgroup; (2) pooled FDR correction across all 197 *P* values to control the overall FDR; and (3) a Bonferroni correction, wherein each individual *P* value was multiplied by the total number of comparisons and capped at 1.0 to yield a conservative threshold for significance. All 3 corrections were reported for each unit to allow the reader to assess the consistency of findings under varying stringency levels. These correction procedures were implemented in MATLAB (MathWorks), and the final results were tabulated to include the original unadjusted *P* values alongside their corrected counterparts.

While Bonferroni correction offers strict control over the family-wise error rate by adjusting for the total number of tests, it is widely recognized as overly conservative, particularly in exploratory analyses involving large numbers of comparisons. In our study, with 156 parallel tests of seasonal periodicity across multiple geographic units, only the most extreme *P* values remained significant under Bonferroni correction. However, this stringency comes at the cost of increased false negatives, potentially obscuring meaningful patterns. To provide a more balanced assessment, we also applied the Benjamini–Hochberg procedure to control the FDR, both within each subgroup and across all groups combined. This approach allows for a controlled proportion of false discoveries while retaining sensitivity to genuine effects. Several locations that did not meet the stringent Bonferroni threshold nonetheless showed robust significance under both within-group and pooled FDR correction. Therefore, in addition to reporting Bonferroni results, we interpret findings that pass FDR criteria as statistically meaningful and potentially informative, particularly in the context of understanding broad seasonal patterns in public search behavior.

Of note, the amplitude of the cosinor model represents the extent of fluctuation around the mean value over the seasonal cycle. A larger amplitude indicates greater variation between the peak and trough of the fitted curve, reflecting a more pronounced and consistent seasonal pattern in the data. Conversely, a small amplitude suggests weaker or less distinct seasonality, whereas (2) the phase of the cosinor model indicates the timing of the peak within the seasonal cycle. It represents the point in the 12-month period at which the fitted curve reaches its maximum value. By converting the phase (typically expressed in radians or degrees) to a calendar reference, we can associate it with a specific month of peak activity. For example, a phase corresponding to $\frac{\pi }{2}$ radians (or 90°) in a 12-month model aligns with April, indicating that the seasonal peak occurs around that time.

The analysis of the cosinor model aims to test two hypotheses: (1) does the public’s interest in “learning about gout” vary throughout the year? (2) Does the public’s interest vary in a way that manifests increased activity in specific months (and, by extension, seasons) during the year, that is, over 12 months. An approximate “peak” activity is assigned to the time representing the maximum of the fitted cosinor model. Formal ethics approval was not required since this work comprised publicly available anonymous data and contained no personally identifiable information.

To assess the possibility of bimodal or asymmetric seasonal patterns, we extended the standard 12-month cosinor model by incorporating an additional 6-month harmonic. Model comparisons indicated no substantial improvement in fit with the inclusion of the second harmonic. Furthermore, the statistical significance of the bimodal term was evaluated using *P* values adjusted by the Benjamini–Hochberg FDR procedure to control for multiple comparisons. The 6-month component did not reach statistical significance under this correction, supporting the use of a single 12-month harmonic model. This choice is further justified by clinical literature, which consistently reports a single seasonal peak for the condition under investigation. We used the difference in Bayesian and Akaike information criterion (Δ AIC) to evaluate whether the bimodal model offered a substantially better fit than the unimodal cosinor model. A positive Bayesian information criterion (ΔBIC) indicates that the bimodal model achieves a better balance of goodness-of-fit and parsimony, with larger values providing stronger justification for the added complexity of modeling 2 seasonal peaks per year. Consistent with standard interpretive thresholds [28], we considered ΔBIC values ≥6 as moderate evidence, and values ≥10 as strong evidence, in favor of the bimodal model. This approach allowed us to systematically identify features for which the added seasonal component improved explanatory power beyond what could be captured by a single annual rhythm. Notably, the results indicated that a bimodal distribution was not warranted in any case, with one exception: the search term **“**obesity,**”** which exhibited 2 distinct peaks and will be discussed in more detail in a subsequent section.

## Results

To assess the robustness of seasonality across multiple geographies and query categories, we applied 3 complementary statistical correction procedures to the cosinor model *P* values: (1) within-group FDR using the Benjamini–Hochberg procedure, (2) pooled FDR across all comparisons, and (3) the conservative Bonferroni correction. As expected, the Bonferroni method yielded the fewest statistically significant results, consistent with its well-known tendency to reduce false positives at the cost of increased false negatives. In contrast, the 2 FDR-based approaches yielded more comparable results, identifying a broader yet still controlled set of significant findings. Importantly, many of the key seasonal signals remained robust across all 3 correction methods, affirming the reliability of the findings. Given the exploratory nature of infodemiologic research, and the inherent correlation structure of the data (eg, geographic and temporal clustering), FDR corrections, especially at the within-group level, offer a more balanced approach, capturing true signals while minimizing type I error inflation. Results for all cases are summarized in Table 1, where for each group (eg, countries using English queries, language-specific countries, US states, US/Canada cities, and symptom-related queries), we report the number of entries with significant seasonality based on four approaches: (1) unadjusted *P* values (*P*<.05); (2) within-group false discovery rate correction; (3) pooled false discovery rate correction across all 197 tests; and (4) Bonferroni correction. While unadjusted and false discovery rate-corrected results yield consistent detection of seasonality, Bonferroni correction, which is more conservative, substantially reduces the number of significant findings. This highlights the trade-off between sensitivity and specificity in multiple comparisons analysis.

**Table 1.** Comparison of the number of statistically significant seasonal patterns in gout-related search queries across 5 groups using different *P* value correction methods.

Group	Number of entries	Original *P* value <.05	Within-group FDR^a^	Pooled FDR	Bonferroni correction
Countries – English	70	41	36	39	7
Countries – Language	20	15	15	15	2
US States	50	49	49	49	9
US/Canada Cities	36	33	33	31	10
Symptoms	21	18	18	17	8

a FDR: false discovery rate

**Figure 1.**
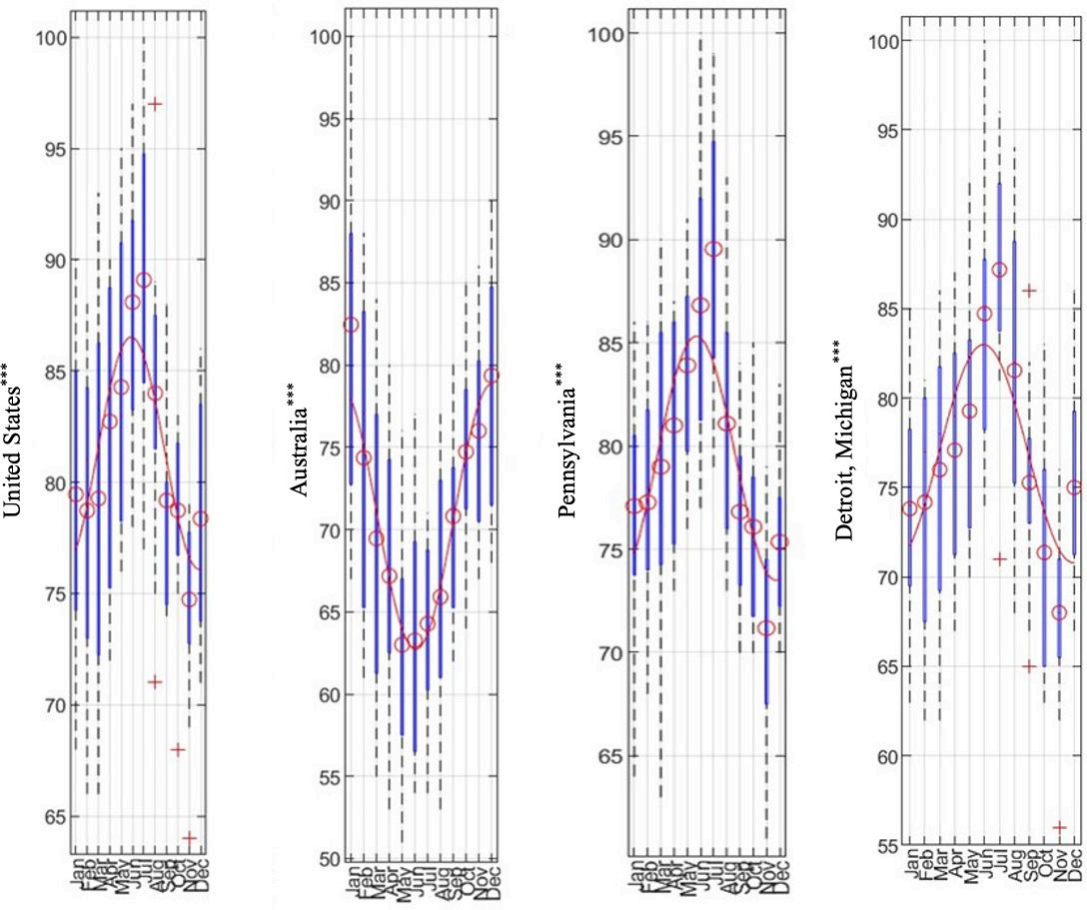
Seasonal variations in gout search interest across regions: line plots with 95% CIs (blue bars) showing monthly trends in search activity for gout-related terms. *** indicate statistically significant seasonal variation.

**Figure 2.**
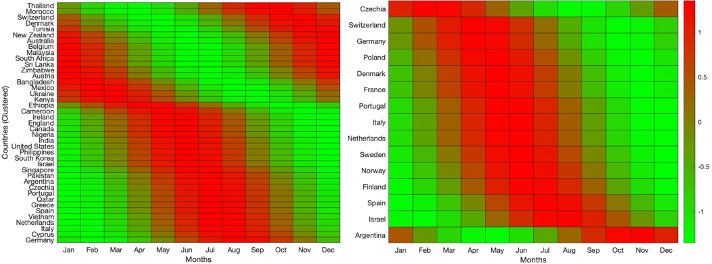
Heatmap showing monthly variations in search interest across countries using the keyword “gout” (left panel). Red indicates peak interest, green indicates low interest. The heatmap on the right reflects interest in the keyword “gout,” translated into language-specific terms, with each country using its dominant language.

We performed a comprehensive inspection to determine countries with adequate data. A final list of 70 countries was analyzed, representing a fair distribution across all continents. For all countries, the cosinor parameters and the confidence in the season-dependent traffic queries are presented in Multimedia Appendix 1 (worksheets All Results and World), while the plots corresponding to the yearly averages for all 70 countries are shown in Multimedia Appendix 2. The 70 countries worldwide represent a wide geographical distribution regarding latitude and are reasonably populated. Typical annual query frequency plots are shown in Figure 1 for the United States and Australia (see MultimediaAppendices 1^4^ for complete results). Notably, the countries exhibited a reversal in the peak of queries. Figure 2 (left panel; for complete results see MultimediaAppendices 1, 2  5) depicts a heatmap showing monthly variations in search interest across countries, with red indicating high interest, whereas green indicates low interest.

Even though English terminology is commonly used, we further elaborated on language-specific terms. We examined searches based on 12 languages: Czech, Danish, Dutch, French, Finnish, German, Greek, Hebrew, Italian, Norwegian, Portuguese, Romanian, Spanish, and Swedish. A total of 20 countries were selected, for which the cosinor parameters and the confidence in the season-dependent traffic queries are presented in Multimedia Appendix 1 (worksheet World language specific). The corresponding yearly averages are depicted in MultimediaAppendices 4  5. Figure 2 (right panel) depicts a heatmap showing monthly variations in search interest across countries, with red indicating high interest, whereas green indicates low interest.

**Figure 3.**
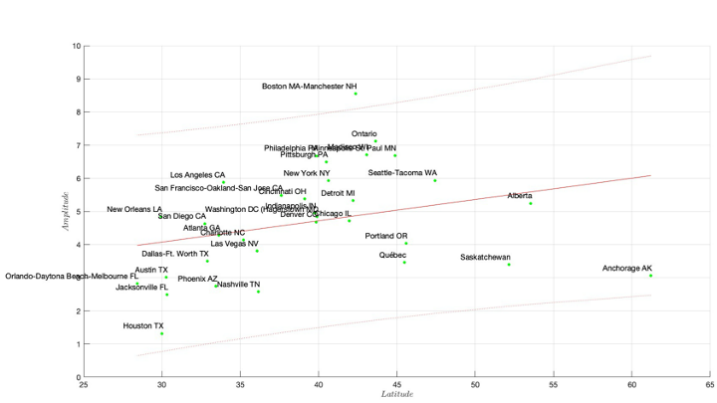
Geographic variation in amplitude of gout-related search trends: scatter plot showing the relationship between latitude and the amplitude of seasonal search patterns for gout-related terms across various cities in North America.

Subsequently, we analyzed “gout” queries in all 50 US states in Multimedia Appendix 1 (worksheet US states), while the corresponding yearly averages are depicted in Multimedia Appendix 3. We further examined query data from 32 major cities in the United States and 4 in Canada, and results are provided in Multimedia Appendix 1 (worksheet US cities) and all characteristic profiles in Multimedia Appendix 4. The cities in Canada were added to better populate the geographic region up to Alaska, with typical results shown in Figure 1 for the state of Pennsylvania. Inspection of the entirety of the data confirms that the Google Trends data demonstrates robust seasonal patterns. A targeted analysis of areas was performed to obtain a more granular view of the peak of gout-related searches in the United States. Further examination of the amplitude of the seasonal oscillations and analysis of the relation between a city’s latitude and the differences (peak to trough) is shown in Figure 3 (detailed results are in MultimediaAppendices 1  4. As seen in the results, linear regression yielded a positive slope (0.0643), but the association was not statistically significant (*P*=.09), and the 95% CI (–0.0113 to 0.1399) included zero, suggesting weak or no linear trend (*R*²=0.0884). The Pearson correlation was also not significant (*r*=0.2974; *P*=.09), whereas the Spearman rank correlation was significant (ρ=0.4405; *P*=.01), indicating a monotonic but not necessarily linear relationship. These results suggest that amplitude may increase with latitude in a nonlinear or ordinal fashion.

**Figure 4.**
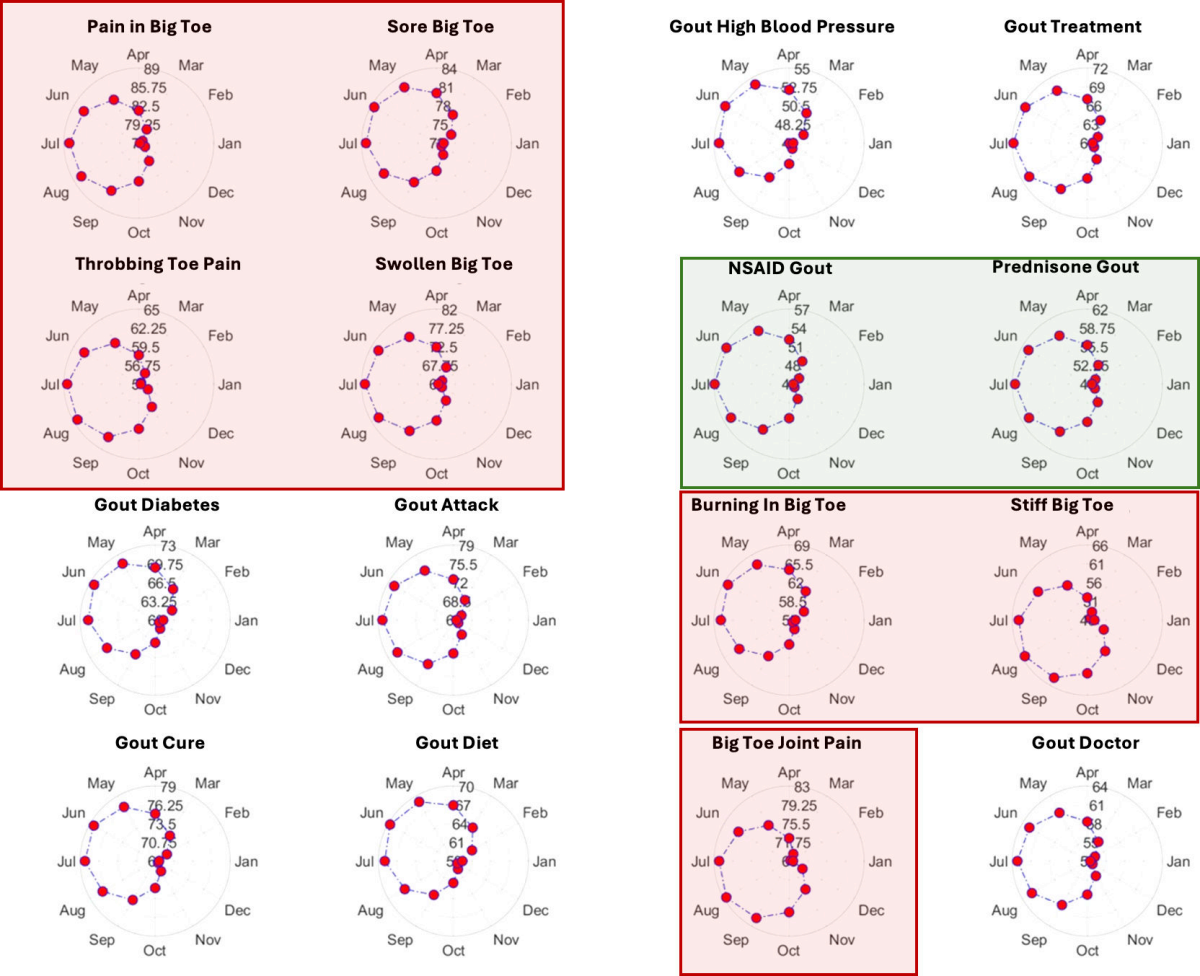
Seasonal trends in gout-related searches: polar plots illustrating monthly patterns in search interest for various gout-related terms. Red-shaded panels focus on symptoms.

Finally, focused only on the United States, we considered searches that either contained descriptions of symptoms without explicitly mentioning the term “gout” or queries that combined the term “gout” with other relevant queries. The terms used were: “burning big toe,” “stiff big toe,” “big toe joint pain,” “pain in big toe,” “sore big toe,” “throbbing toe pain,” “swollen big toe,” “gout doctor,” “gout diabetes,” “gout attack,” “gout cure,” “gout diet,” “gout symptoms,” “gout high blood pressure,” “gout treatment,” “NSAID gout,” and “prednisone gout.” The polar plots indicating strong seasonal patterns of behavior are depicted in Figure 4 (see MultimediaAppendices 1  6 for detailed results).

## Discussion

### Principal Findings

This study confirms that public interest in gout, as measured by Google Trends, exhibits strong and recurrent seasonal patterns that align closely with the established clinical seasonality of gout flares. Analysis of over a decade of search data across 70 countries, all US states, and major North American metropolitan areas revealed consistent peaks in search activity during the late spring to early summer months in the northern hemisphere and in late fall to early winter in the southern hemisphere. The cosinor modeling identified significant seasonal rhythmicity in 50% of the countries analyzed and over 90% of US states and cities, underscoring the robustness of these trends. Furthermore, symptom-based and treatment-related queries demonstrated parallel seasonal behaviors, while language-specific search terms revealed that English queries often produced clearer seasonal signatures, particularly in bilingual or digitally literate populations. Together, these findings support the hypothesis that digital search behavior reflects real-world disease dynamics and suggest that infodemiology offers a scalable and timely proxy for studying the seasonal dimension of gout.

Seasonality is a complex concept. A simplistic approach equates seasons with the earth’s position relative to the sun: summer closer, winter further; hence longer, warmer days. However, seasonality is far more complex than that. In addition to temperature, several other factors contribute: climate, dietary shifts based on food availability, activity levels, and exposome, to name a few. Furthermore, geographic location significantly impacts seasonal characteristics: latitude, altitude, proximity to water, local weather patterns (influenced by currents, mountains, etc). Furthermore, expressing an interest in learning about something (in this case, quantified by the volume of queries regarding “gout”) does not imply either causality or define a clear, quantifiable marker of behavior. This behavioral information can only be used as corroborating evidence to support a more thorough and complete follow-up analysis; however, in general, information-seeking behaviors are driven by the desire to learn about something specific [29]. Therefore, the present work aims to determine the emergence of regularity in human behavior and examine whether it correlates qualitatively with observations based on well-defined markers. When assembled and interpreted with care, human behavior, expressed by activities that reflect public interest in specific topics, can define independent observation. Of course, this type of information is hardly unbiased as it depends not only on technical limitations (having access to data sources) but also on complex, usually education/socioeconomic factors and barriers, which determine how individuals access information sources, which nowadays are, almost exclusively, internet-based. Nevertheless, when interpreted within the appropriate context, monitoring Google search activity offers a valuable and timely lens through which to detect emerging seasonal patterns in disease-related interest, providing complementary insights that may precede or support more traditional epidemiological indicators.

Understanding these seasonal dynamics has profound implications for public health. For instance, patterns in online search behavior offer valuable insights into how individuals respond to seasonal changes in disease prevalence, symptom flare-ups, and preventive health measures. By analyzing these digital behaviors, health care professionals and researchers can identify public perceptions, knowledge gaps, and behavioral trends affecting understanding and managing chronic diseases and seasonal health challenges. Integrating environmental seasonality into health communication strategies provides a powerful framework for enhancing disease prevention and management. By aligning interventions and awareness campaigns with seasonal trends, health care providers can optimize their efforts to address health challenges and improve outcomes throughout the year.

Internet searches reveal the public’s most common concerns and misconceptions about diseases, symptoms, and treatments [30]. Understanding this data can guide tailored public health campaigns that address specific misconceptions, provide clear and actionable health information, and promote disease prevention strategies effectively, thereby improving overall health literacy [31]. Monitoring internet searches also plays a critical role in pharmacovigilance. Patients frequently search for information about the side effects of medications. A high volume of searches concerning a specific treatment’s adverse effects could signal the need for closer monitoring and potentially guide health care providers in offering safer medication alternatives. While Google Trends should not be used in isolation, it provides a real-time, scalable source of behavioral information that, when combined with other data, enables the design of more effective, patient-centered interventions [32].

### Gout Across the Globe

We developed a global view of gout-related search behavior by collecting Google Trends data from 70 countries representing a substantial portion of the world, given that 193 nations are members of the United Nations [33]. The countries included in our analysis span all continents and are illustrated in Figure 2. While we aimed for broad coverage, several countries had limited, if any, usable data. This could stem from various factors, including limited access to internet resources, poor health care infrastructure, low levels of digital literacy, or high levels of poverty. Nonetheless, internet availability was generally sufficient across the countries analyzed; according to 2021 World Bank data, the average internet penetration among the 70 countries was 80%, with a median of 88% (see Multimedia Appendix 1).

Among the 70 countries analyzed using English-language search terms, 41 exhibited statistically significant seasonal variation based on unadjusted *P* values. Applying within-group FDR correction slightly reduced this number to 36, while the pooled FDR correction preserved much of the original significance, with 39 entries remaining significant. However, the Bonferroni correction, being the most conservative, identified only 7 significant cases. These results suggest a strong and widespread seasonal signal in English-language searches, though interpretation depends on the chosen correction method (Table 1). Some countries, such as Russia, China, Brazil, and Argentina, are geographically vast and internally diverse. As such, assigning a single seasonality descriptor to an entire nation may obscure important regional differences. Intracountry variability likely contributes to cases where no clear seasonal trend was observed. In our analysis, each country was treated as a single unit to provide a general overview of its seasonal search behavior. Multimedia Appendix 1 summarizes the demographic and geographic characteristics of the 70 nations analyzed.

Using cosinor modeling, we assessed seasonal variation in search interest and categorized countries into 2 groups based on the presence or absence of statistically significant seasonality. One group exhibited clear seasonal variation in gout-related queries, while the other showed relatively uniform interest throughout the year. For example, as shown in Figure 1, countries such as Australia and the United States demonstrated consistent and robust seasonal trends. These patterns are marked by peaks and troughs that align with known clinical exacerbations of gout, particularly in the late spring to early summer months.

Importantly, our findings reflect previously reported patterns. A previous study [23,34] observed statistically significant seasonal variation in gout-related search activity in the United States, United Kingdom, Canada, Ireland, Australia, and New Zealand, with northern hemisphere countries peaking in late spring to early summer, and southern hemisphere countries showing peaks in November and December. Our study expands on this work by incorporating a broader geographic scope and highlighting consistent seasonal trends not only in national data but also at more localized levels, such as US states (eg, Pennsylvania) and metropolitan areas (eg, Detroit).

Furthermore, our analysis underscores the influence of geographic position and climate on the timing and amplitude of seasonal patterns. These hemispheric differences, illustrated in Figure 2, reinforce the role of environmental and climatic factors in shaping public interest in gout and potentially reflect true seasonal variation in symptom presentation. The stark contrast between months highlights how search behavior correlates with local climate conditions. Hemispheric differences are evident, with northern hemisphere countries like the United States and Canada experiencing peaks during colder months, while southern hemisphere nations such as Australia and New Zealand see higher activity during their cooler seasons. This hemispheric contrast underscores a potential link between lower temperatures and increased gout incidence, possibly due to seasonal dietary changes or physiological stressors that exacerbate symptoms. In addition, the heatmap reflects language-specific searches, which may introduce

### Language-Dependent Global Analysis

In the 20 countries where searches were conducted using language-specific terms (eg, “gota” for Portuguese), 15 showed significant seasonality across all correction methods except Bonferroni. While both the within-group and pooled FDR corrections retained the full set of 15, the Bonferroni correction reduced this number to just 2. The consistency between unadjusted and FDR-corrected results suggests the seasonality signal is robust, though relatively weaker or more variable than in English-language searches (see Table 1). Gout flares are known to be season-dependent and peak during the (relative) late spring-early summer. Although “gout” is a universally recognized keyword for the disease, a behavioral analysis must account for the language-specific nuances of Google search queries. The comparison of the heatmaps in Figure 2, but more importantly, in Multimedia Appendix 1 (worksheet World language specific) further elucidates the global patterns of seasonal search interest.

In some cases, search queries using English medical terms may exhibit more robust or distinct seasonal patterns than equivalent terms in the local language; in others, not. In Austria, Brazil, Greece, Italy, Mexico, and Portugal, the use of German, Portuguese, Greek, Italian, and Spanish seems to produce stronger signals than the searches in English. Such discrepancies can arise from differences in user behavior, digital literacy, and the broader accessibility of health information online. English is often perceived as the language of science and medicine, and as such, users may turn to English terms when seeking detailed, authoritative content, particularly when researching symptoms, conditions, or treatments. It is common for individuals in non-English–speaking countries to conduct health-related searches in English for a variety of reasons. First, English dominates scientific and medical discourse, making it the preferred language for medical professionals, students, and health-literate individuals who wish to access global resources such as PubMed, Mayo Clinic, or WebMD. Second, English-language queries frequently return more comprehensive or higher-quality results, reinforcing the tendency to use English over local terminology. In bilingual or English-proficient regions, such as Israel and parts of Europe, this behavior is even more prevalent, especially among urban populations and academic users. Moreover, the choice to search in English may reflect a desire for precision, especially when native-language terms are ambiguous, have multiple meanings, or are less medically standardized. Collectively, these factors help explain why English search terms may yield clearer or more reliable patterns in Google Trends data, and they highlight the importance of incorporating both English and local-language keywords in any comprehensive analysis of health information-seeking behavior.

### Gout Searches Across North America

The results from the 50 US states displayed overwhelming consistency and robustness. All but 1 state exhibited statistically significant seasonality using unadjusted *P* values, and this level of significance was fully preserved across both FDR corrections. Even under Bonferroni correction, 9 states retained statistical significance, reinforcing the strength of the seasonal trend in gout-related search activity across the United States (Table 1). According to recent US census data [35], internet use in US households has grown considerably. In 2018, it was reported that 92% of households had at least 1 type of computer, and 85% had a broadband internet subscription. In addition, smartphone ownership was the most common in 84% of households. Urban households were generally more likely to use computing devices and have internet subscriptions than rural households. Thus, technology is not a barrier in the United States. Despite its size, the United States appears more homogeneous compared to continents like Asia or Africa, where the variance in economic development, urbanization, and lifestyle can be more pronounced across different countries or regions within the same country.

Our state-wide analysis revealed consistent seasonal variation, with peaks appearing in the spring/summer seasons (see Multimedia Appendix 3); New York, New Jersey, Pennsylvania, Vermont, and others exhibit significant variations. In contrast, Arkansas, Arizona, Florida, Hawaii, Montana, North Dakota, New Mexico, Nevada, and South Dakota exhibit a less pronounced seasonality. This result could be a combination of reasons, from local climate (Arkansas, Florida, and Hawaii) to sparseness of data (Montana, North Dakota, and New Mexico). A weak correlation exists between the amplitude of the fitted cosinor and latitude (Multimedia Appendix 1, worksheet US states).

Of the 36 cities analyzed in the United States and Canada, 33 showed significant seasonality using the unadjusted *P* value threshold. This strong signal was confirmed by both FDR correction methods, which preserved significance in 33 and 31 cities, respectively. Bonferroni correction, while more restrictive, still identified 10 cities with significant seasonal patterns, Table 1. These results underscore the prevalence of city-level seasonality in public interest related to gout, especially in urban centers. Data from 32 major metropolitan areas in the United States and 4 in Canada mirror those obtained from the analysis of US states (see MultimediaAppendices 3  4). Figure 3 explores the relationship between latitude and the amplitude of seasonal variations in gout-related searches. Qualitatively, as we move north, the severity of gout across seasons appears more pronounced. Figure 3 reveals a general trend of increasing amplitude with increasing latitude. A linear regression analysis examining the association between peak month and latitude yielded a positive slope (0.0643), but this relationship was not statistically significant (*P*=.09), and the 95% CI for the slope included zero (–0.0113 to 0.1399), indicating substantial uncertainty. The R² value was modest (0.0884), suggesting that latitude accounted for less than 10% of the variance in peak timing. Similarly, the Pearson correlation coefficient (*r*=0.2974) did not reach statistical significance (*P*=.09), and its confidence interval crossed zero. However, the Spearman rank correlation was statistically significant (ρ=0.4405; *P*=.01), with a 95% CI that excluded zero (0.1145 to 0.6809), indicating a significant monotonic relationship. Together, these findings suggest that while a strict linear relationship may be weak or inconsistent, there is evidence of a nonparametric, ordinal association between latitude and peak timing, warranting further investigation into potentially nonlinear or threshold effects.

Seasonality, latitude, and prevalence of disorders are subjects of contention in the literature [36-38]. Higher latitudes experience more extreme temperature variations, which may influence behaviors and physiological factors associated with gout exacerbation. Alternatively, dietary patterns that shift with seasons, such as higher purine intake during winter holidays, could be contributing factors. Our results suggest that regions with more pronounced seasonal differences in temperature and climate potentially exhibit greater seasonal fluctuations in search interest; however, the hypothesis definitely needs further evaluation.

### Searching for Gout-Related Terms

For the 21 gout-related symptom terms, 18 were found to be significant with unadjusted *P* values. Both within-group and pooled FDR corrections preserved nearly all these signals (18 and 17, respectively), while Bonferroni correction identified 8 as statistically significant. These findings indicate that symptom-based search behavior also follows a distinct seasonal pattern, aligning well with known clinical features of gout flares (Table 1). The polar plots in Figure 4 offer a detailed categorization of seasonal trends in gout-related online searches in the United States, grouped into 3 main areas: symptoms, treatments, and broader management strategies. Symptom-related searches, such as “NSAID Gout” and “Prednisone Gout,” exhibit parallel seasonal patterns, suggesting these searches are a reactive behavior to acute gout flares. In addition, searches for more specific gout-related terms such as “gout attack,” “gout flare,” “gout doctor,” “gout cure,” and “gout treatment,” also show strong seasonality, consistent with the documented spring-summer exacerbation of gout symptoms.

Interestingly, the likelihood of seasonal variation in treatment-related terms like “prednisone” and “NSAID” depends on their context. When these terms are searched in isolation, no significant seasonal variation is observed (see Multimedia Appendix 1, worksheet Symptoms, for detailed results). However, their seasonal relevance becomes apparent when coupled with “gout,” underscoring how public behavior, as captured by Google Trends, can reveal nuanced insights into the seasonality of health conditions. In contrast, searches associated with broader management strategies, such as “gout Diet” and “gout Diabetes,” show a more consistent baseline interest throughout the year, with less pronounced seasonality. This contrast highlights the multifactorial nature of health-seeking behaviors, where acute needs drive seasonal trends, while chronic management concerns maintain steady interest over time.

The study confirms the seasonality of gout-related search behavior, demonstrating distinct seasonal patterns in public interest as reflected in Google search queries. These patterns align closely with the clinical seasonality of gout flares, peaking in late spring to early summer in the northern hemisphere, while the southern hemisphere shows reversed trends due to opposing seasonal cycles. This geographic variability in gout-related search interest highlights the influence of climate and geography on public health behavior. Furthermore, analyzing language-specific queries reinforced the observed seasonal patterns, emphasizing the need to account for linguistic diversity in infodemiology studies. Notably, searches for gout symptoms using generic terms such as “burning big toe” or “swollen big toe,” without explicitly mentioning “gout,” followed similar seasonal trends, underscoring the alignment between public health information-seeking behavior and the clinical characteristics of the disease.

To further investigate whether a bimodal seasonal pattern provided a better fit than the standard unimodal cosinor model, we calculated the difference in BIC $\Delta BIC=BIC_{uniM}-BIC_{biM},BIC=kln\left(n\right)-2\ln\left(SSE\right),k=3unimodal,5bimodal,n=\#ofobservations$, restricting interpretation to terms where the bimodal model’s seasonal component was statistically significant (*P*<.05). Entries for which neither the unimodal nor bimodal model reached significance were excluded from comparison (denoted as ΔBIC=-999). Among the remaining terms, most exhibited ΔBIC values <6, indicating only weak or negligible support for the more complex bimodal structure. However, a subset of terms showed stronger evidence. Notably, “obesity” (ΔBIC=11.46) and “swollen big toe” (ΔBIC=12.48) exceeded the conventional threshold of ΔBIC≥10, providing strong evidence in favor of the bimodal model. In addition, “gout Attack” (ΔBIC=7.30), “Pain Big Toe” (ΔBIC=7.18), and “Prednisone Gout” (ΔBIC=6.00) fell within the moderate evidence range. The bimodal pattern observed for **“**obesity**”** may reflect well-documented cyclical behavior in weight-related interest, with peaks typically occurring in early spring (associated with New Year’s resolutions and presummer health goals) and a second peak in autumn, potentially driven by increased health messaging during insurance enrollment periods or post-summer health resets. Google searches for “obesity” in the United States show 2 annual peaks, April and October, likely reflecting seasonal trends in health awareness, behavioral motivation, and institutional activity rather than actual changes in prevalence. The April peak aligns with renewed interest in fitness and dieting after winter, amplified by World Obesity Day (March 4) and related campaigns. The October peak coincides with pre-holiday health concerns, open enrollment for US health insurance plans, and academic programming. Media releases during both periods further boost public engagement. Together, these factors likely explain the biannual surges in obesity-related search activity seen in Google Trends. In contrast, the bimodality observed in **“**swollen big toe,**”** a symptom closely associated with acute gout attacks, may correspond to known seasonal flare patterns, with peaks in both winter and summer months. These periods have been linked to lifestyle and dietary triggers such as holiday indulgences and dehydration, respectively. While most terms were adequately described by a single seasonal cycle, these findings suggest that specific symptoms or conditions may follow more complex, contextually driven seasonal rhythms.

As a complementary measure to BIC, we also computed the difference in Akaike Information Criterion ($\Delta AIC=AIC_{uniM}\;-\;AIC_{biM},\;AIC=2k-2\ln\left(SSE\right),\;k=3\;unimodal,\;5\;bimodal$) between the unimodal and bimodal models to assess model preference. A positive $\Delta AIC(AIC_{uniM}-AIC_{biM})$ indicates a better fit for the bimodal model, with values>4 representing moderate evidence and values >10 considered strong. To guard against overfitting, only cases with a statistically significant bimodal seasonal term (*P*<.05) were considered. Notably, “obesity” and “swollen big toe” also showed strong ΔAIC values (>10), reinforcing the BIC-based conclusion that these search terms follow a more complex seasonal rhythm best described by a bimodal model.

### Limitations and Broader Concerns

#### Overview

Google Trends, or any other platform monitoring human behavior, offers unique insights and can be a valuable tool for public health surveillance and research when combined with traditional epidemiological methods and other data sources. Understanding its limitations and dangers helps make informed decisions about interpreting and using the data effectively. These include, but are not limited to, the following.

#### Representation

Google Trends, or any similar repository of behaviors, reflects only the actions of people who use Google to search for information. This data may not represent the entire population, especially in regions or among demographic groups with lower internet penetration or a preference for other outlets. Thus, the data may exhibit biases related to age, socioeconomic status, geography, and digital literacy.

#### Aggregation

Trend data is anonymized and aggregated. While this is crucial for user privacy, it also means that data cannot be broken down by critical demographic factors like age, sex, and other health-relevant specifics unless these are explicitly included in the search terms themselves. This lack of detailed demographic data can limit the ability to tailor public health responses to specific populations.

#### Context

Search queries lack contextual depth. For instance, a spike in searches for “flu symptoms” could be driven by increased flu cases or media coverage. Distinguishing between these motivations without additional data sources is challenging.

#### Lack of Standardization

Different individuals use different terms to describe similar symptoms or health concerns, and the popularity of specific terms can change over time. This variability can complicate the interpretation of trends and requires constant adjustments to the search terms used for monitoring.

#### Reliability

The methodology by which Google, or any other social medium, aggregates and anonymizes search data is not fully transparent, which can raise questions about the reliability and accuracy of the trends. Google also periodically updates its algorithms and databases, which can affect historical data comparisons.

#### Overinterpretation

There is a risk of overemphasizing the significance of fluctuations in search data. Not every increase or decrease in search volume may be meaningful, and distinguishing statistically significant trends from normal variability can be challenging without robust analytical tools.

#### Ethical Concerns

Aside from the implications of misinterpretation of data and their analysis, several critical ethical issues emerge. Recording and analyzing human behavior undoubtedly provides significant potential advantages, enabling a better understanding of public interest and emerging needs. However, although individual data is anonymized, group dynamics can be revealed by focusing on targeted geographic areas; second, segments of the population can be targeted and manipulated, while, at the same time, groups can be mischaracterized based on misinterpretation or biased interpretation of data. Newer technologies, primarily associated with wearable devices, promise to revolutionize how we understand human behavior. Therefore, while the benefits are plentiful, we must consider the negative implications.

### Conclusions

Google Trends emerges as a valuable tool for monitoring public interest in chronic diseases such as gout, offering real-time, scalable insights that reflect population-level behaviors. While it is not a substitute for traditional epidemiological surveillance, it serves as a powerful complementary resource that can inform public health communication strategies, disease forecasting, and personalized care planning. This study highlights the strong and recurrent seasonal component of gout-related search activity, demonstrating how public interest aligns with established clinical patterns of disease exacerbation. Moreover, it captures the geographic and linguistic diversity of health-seeking behaviors, illustrating how local climates, cultural practices, and language use influence the way individuals engage with health information online. The broader implications of these findings extend to multiple domains. Understanding the seasonality of inflammatory diseases like gout presents significant opportunities for enhancing patient care and public health planning. For clinicians, anticipating seasonal trends can support more proactive care, enabling timely medication adjustments, patient counseling, and lifestyle recommendations tailored to periods of heightened risk. For public health practitioners, the results underscore the potential of digital behavior data to guide the timing and targeting of awareness campaigns, resource allocation, and prevention strategies. Seasonal surges in search activity may serve as early indicators of increased disease burden, offering an opportunity to intervene before clinical demand escalates. At the policy level, integrating infodemiology into surveillance systems can enhance the responsiveness and precision of health interventions, particularly in the face of climate change and shifting population behaviors. Future research should build on this work by exploring additional contributing factors such as dietary patterns, urbanization, health care access, and socioeconomic disparities, as well as expanding to other inflammatory or environmentally sensitive conditions. Together, such efforts can advance a more holistic and dynamic understanding of health seasonality, leading to more equitable and effective health systems.

## Supplementary material

10.2196/75415 Multimedia Appendix 1 Tables with all results presented in the manuscript.

10.2196/75415 Multimedia Appendix 2 Seasonal distribution of Google searches using the English keyword “gout” across the world.

10.2196/75415 Multimedia Appendix 3 Seasonal distribution of Google searches using the English keyword “gout” across US states.

10.2196/75415 Multimedia Appendix 4 Seasonal distribution of Google searches using the English keyword “gout” across United States and Canada major metropolitan areas.

10.2196/75415 Multimedia Appendix 5 Seasonal distribution of Google searches using language-specific keywords for “gout.”

10.2196/75415 Multimedia Appendix 6 Season distribution of Google searches using gout-related keywords.

## Abbreviations

AIC: Akaike Information Criterion
BIC: Bayesian Information Criterion
FDR: false discovery rate
NSAID: nonsteroidal anti-inflammatory drug
