# Supplementary figures and images for: Seasonal Variation in Public Interest in Gout: Longitudinal Infodemiology Study Using Google Trends (2014–2024)
Source: J Med Internet Res. 2025 Aug 27;27:e75415. doi: 10.2196/75415 (PMC12387374; doi:10.2196/75415)

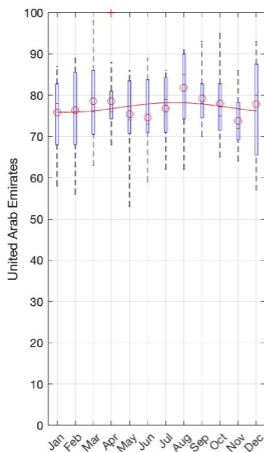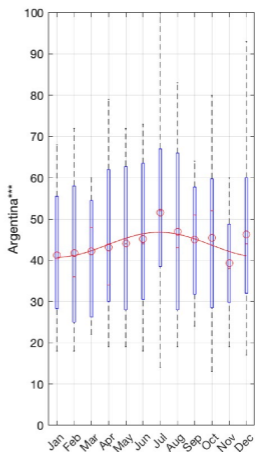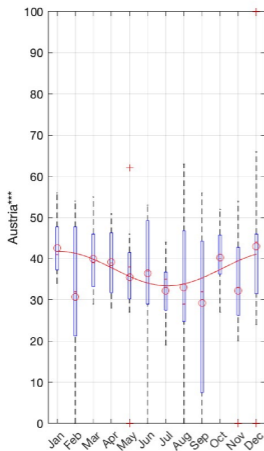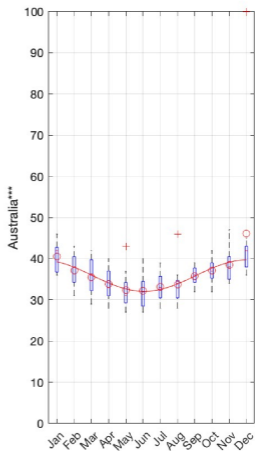

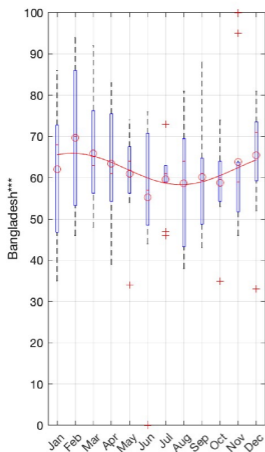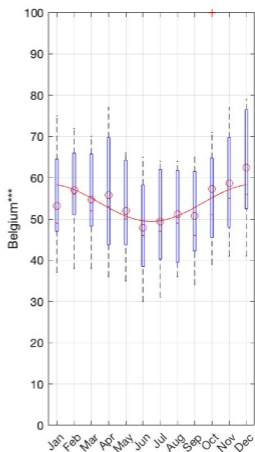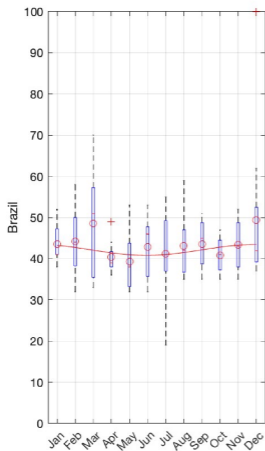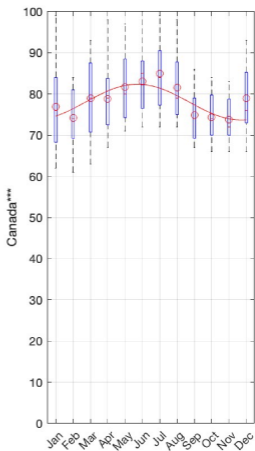

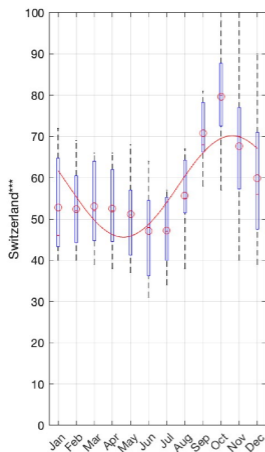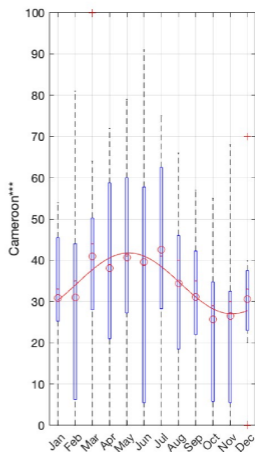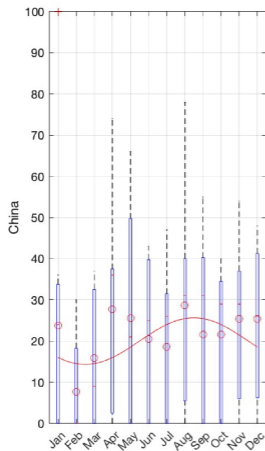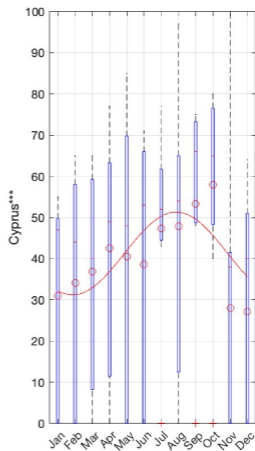

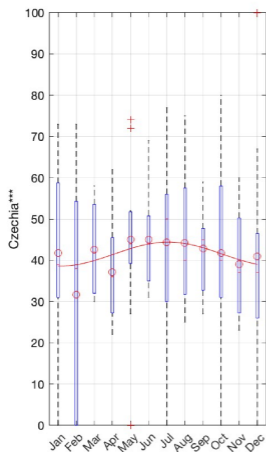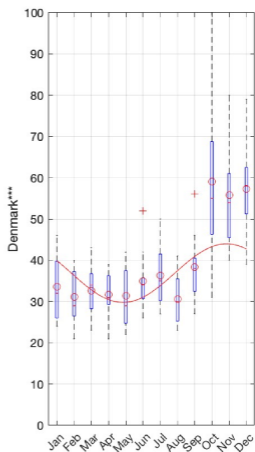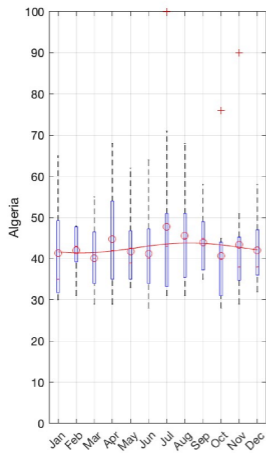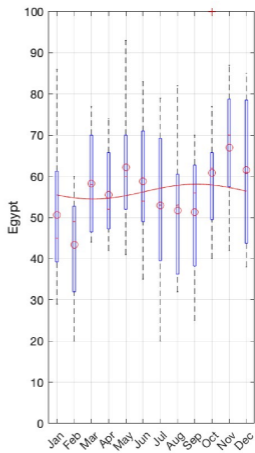

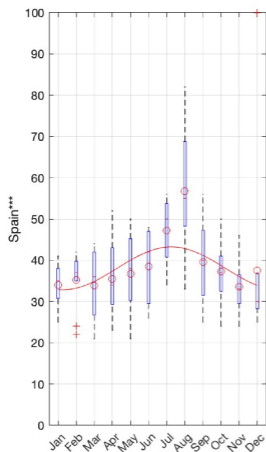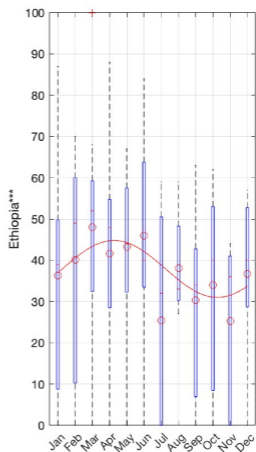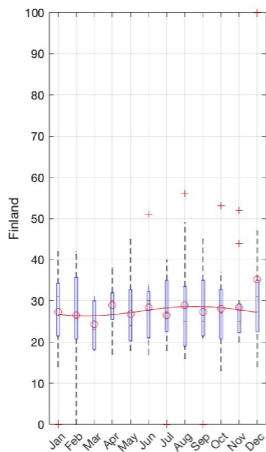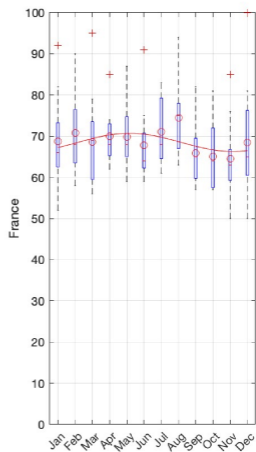

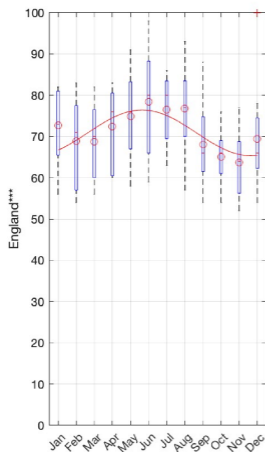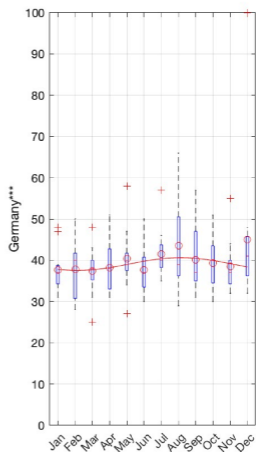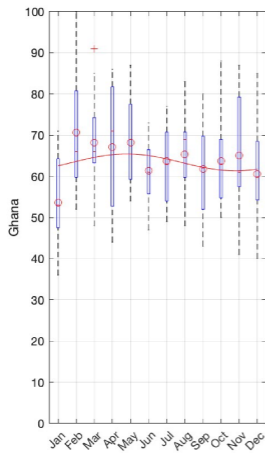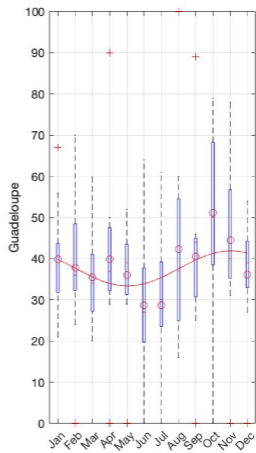

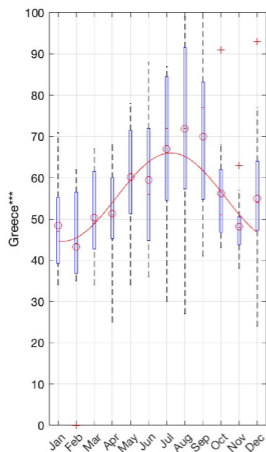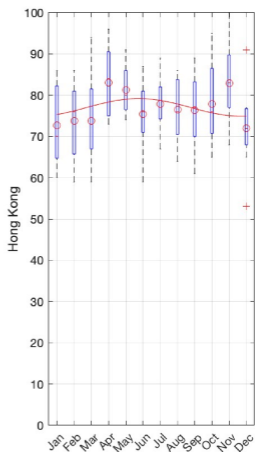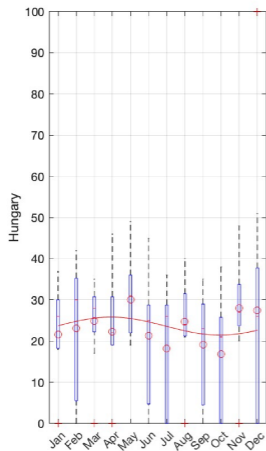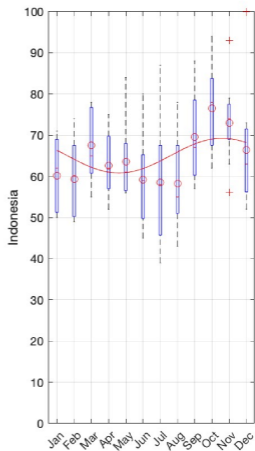

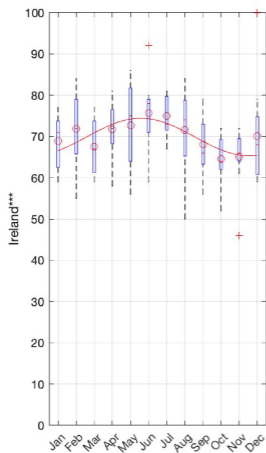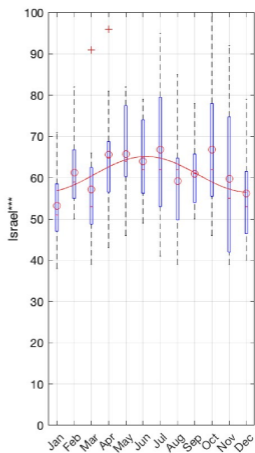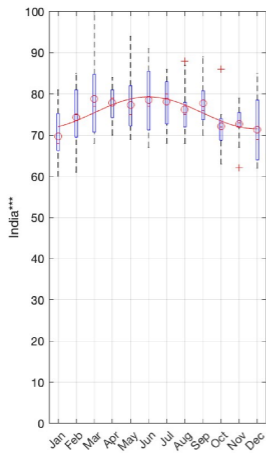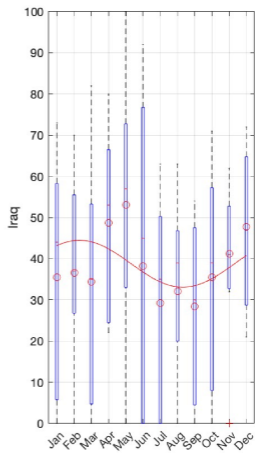

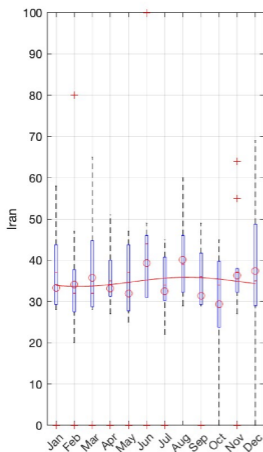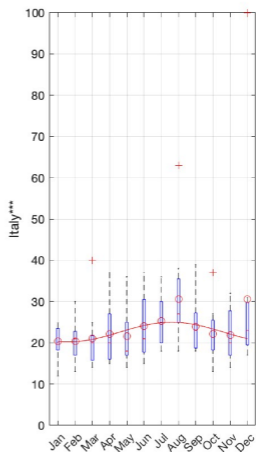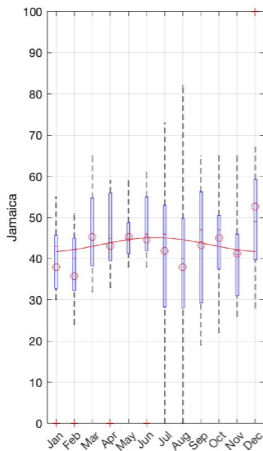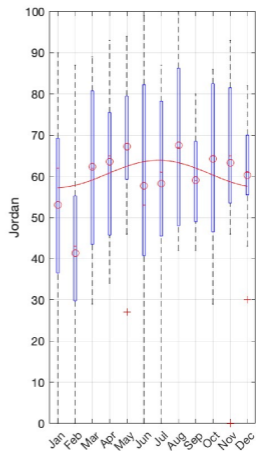

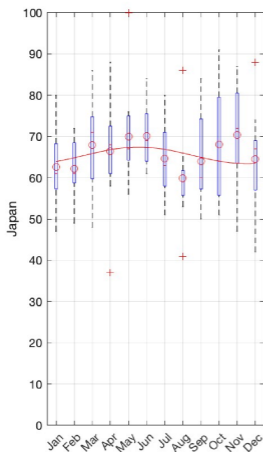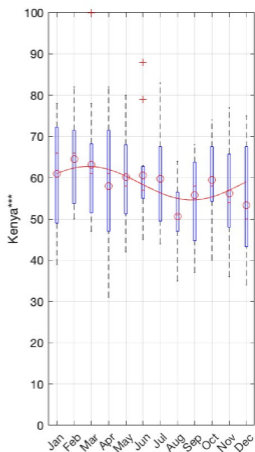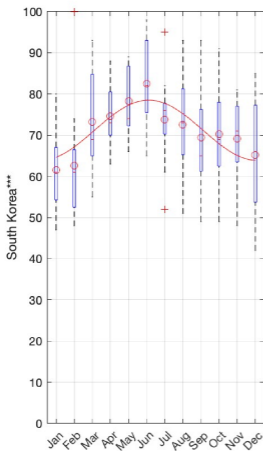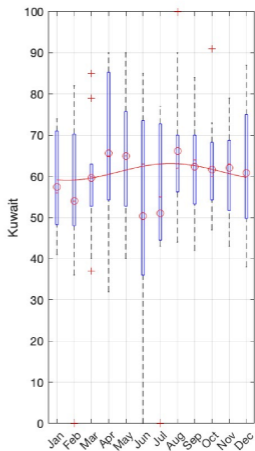

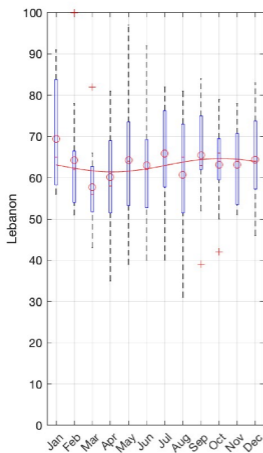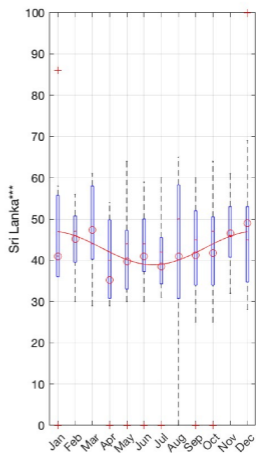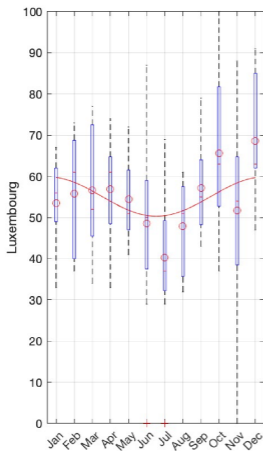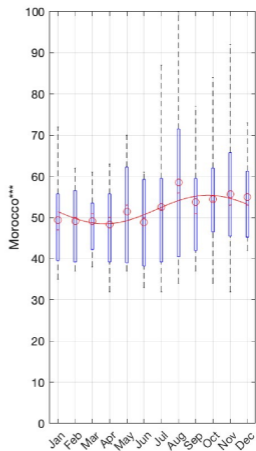

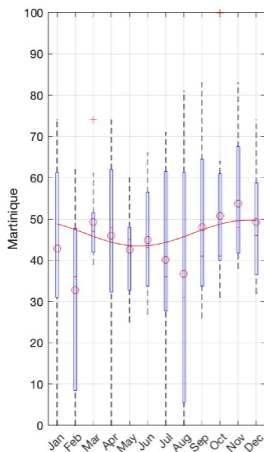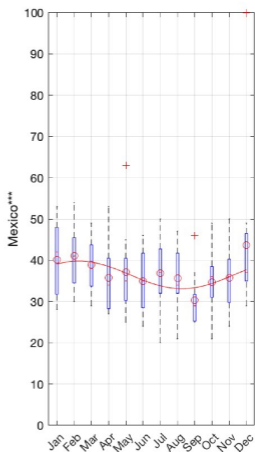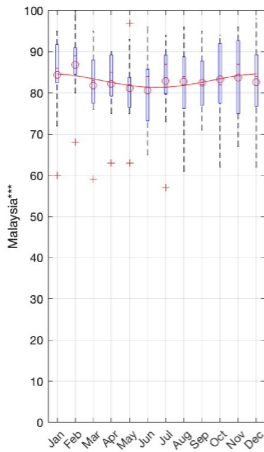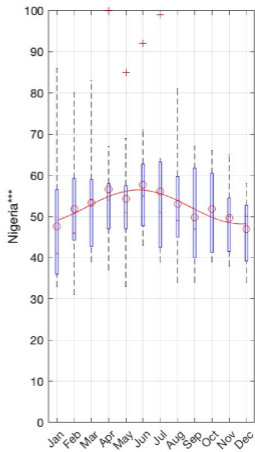

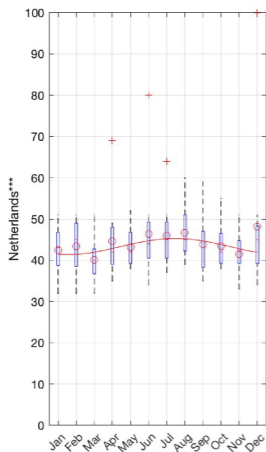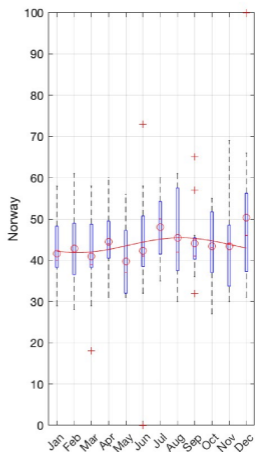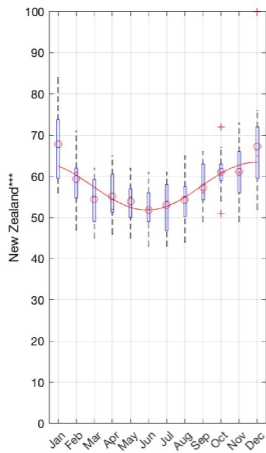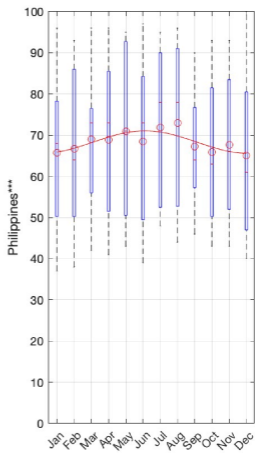

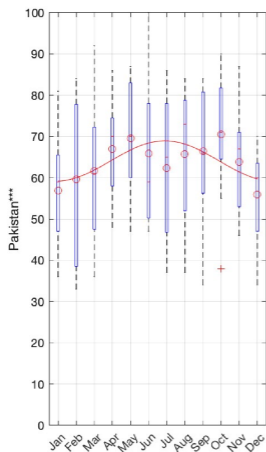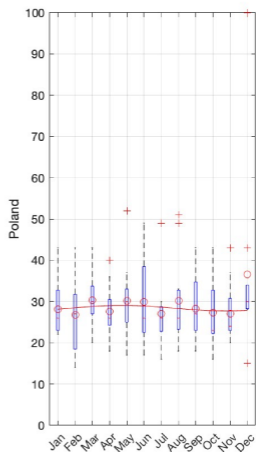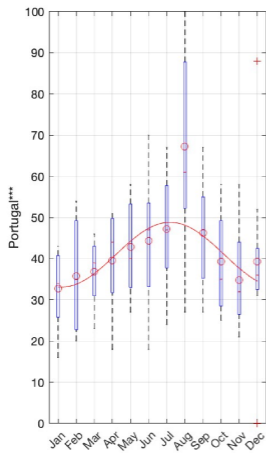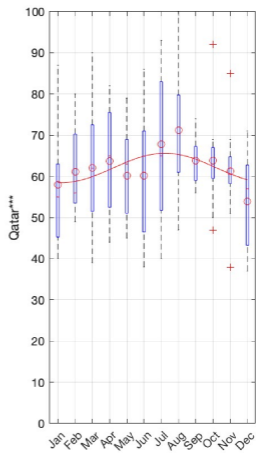

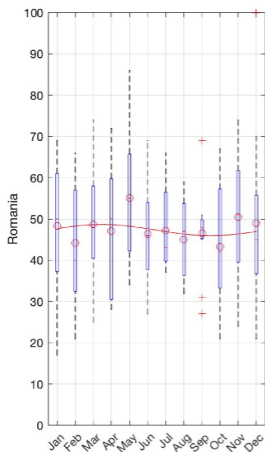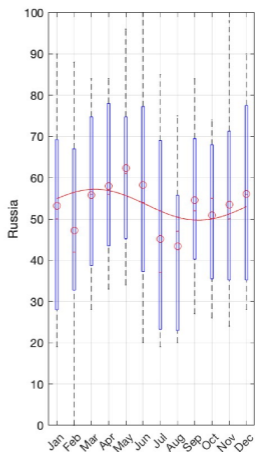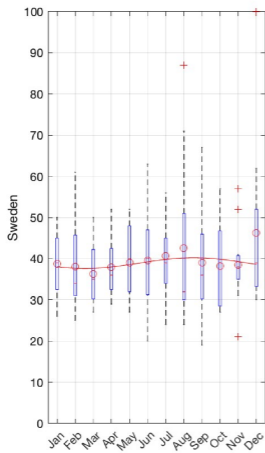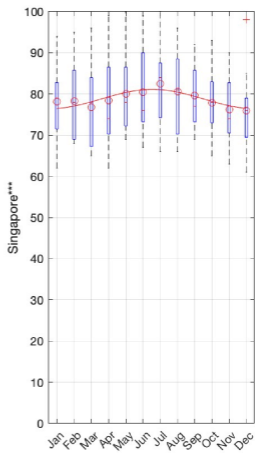

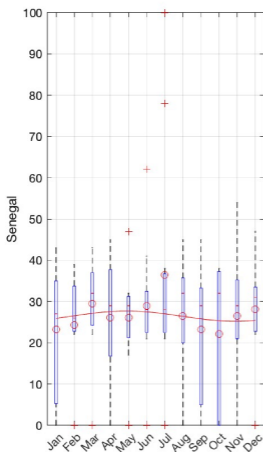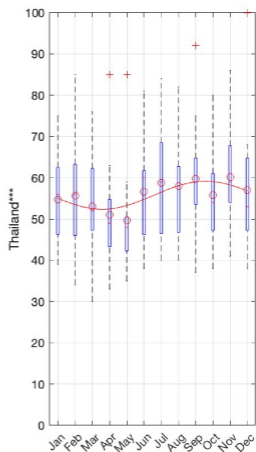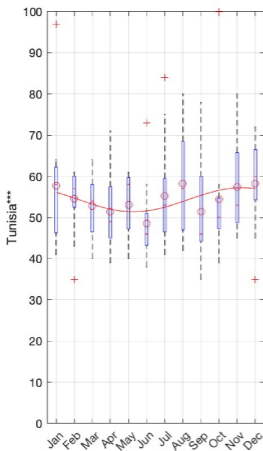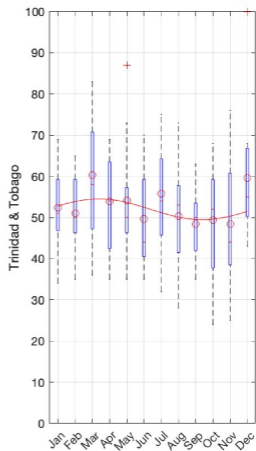

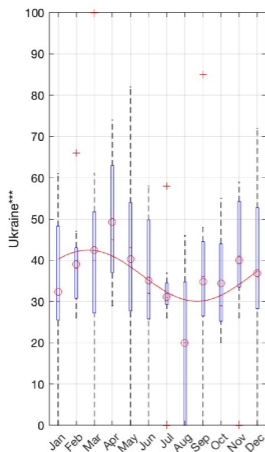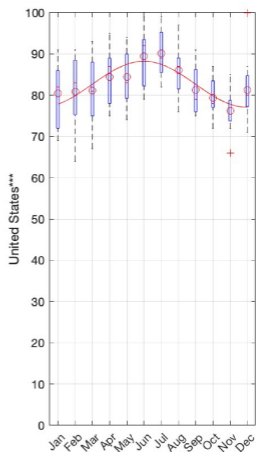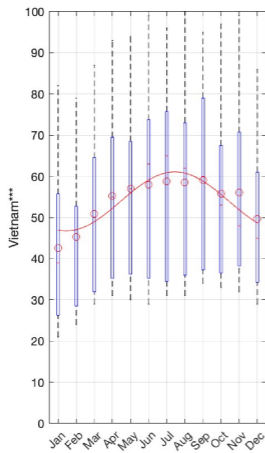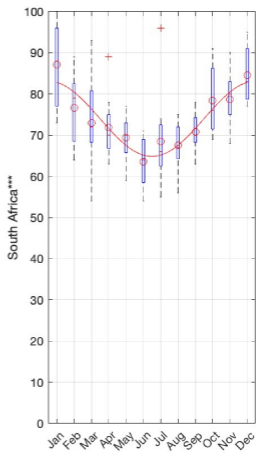

Supplement: Multimedia Appendix 2 [file jmir-v27-e75415-s002.pdf]

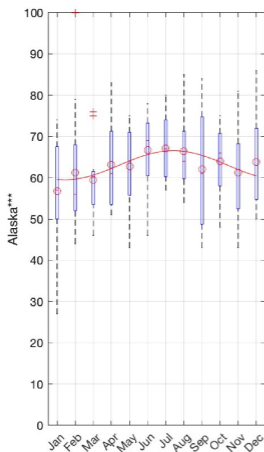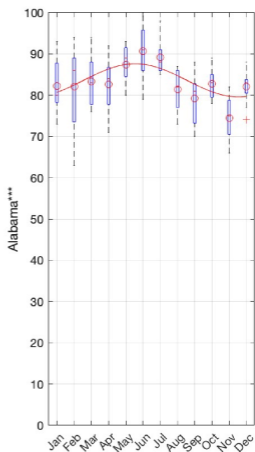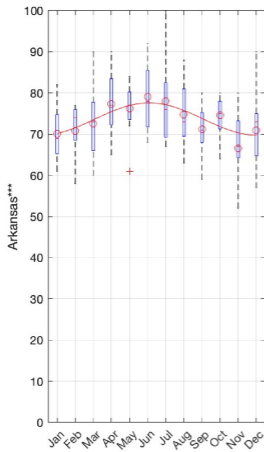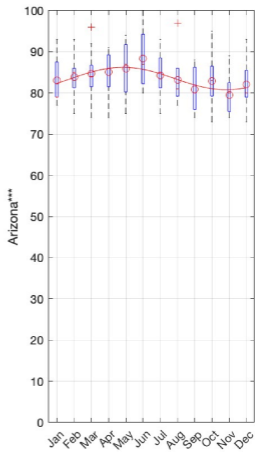

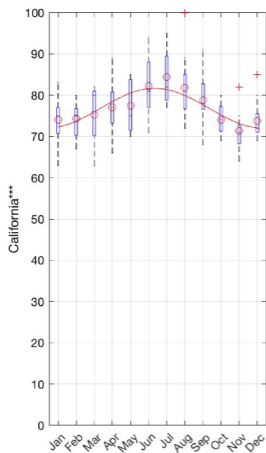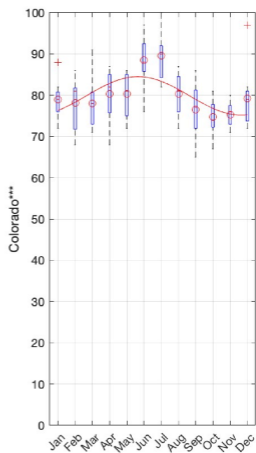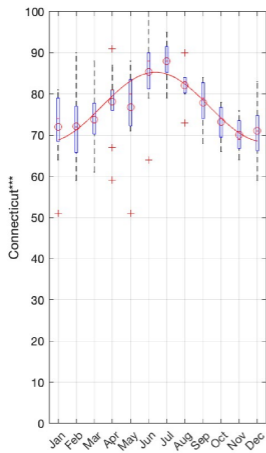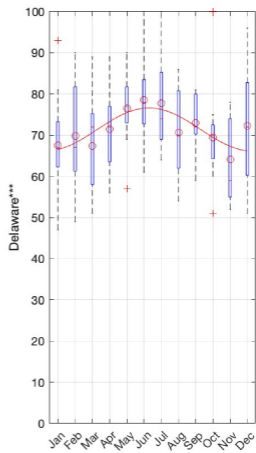

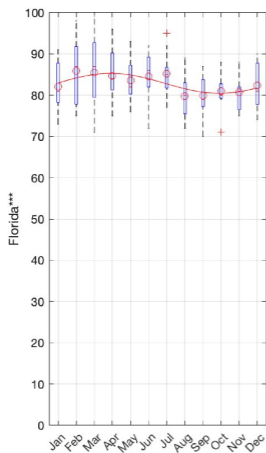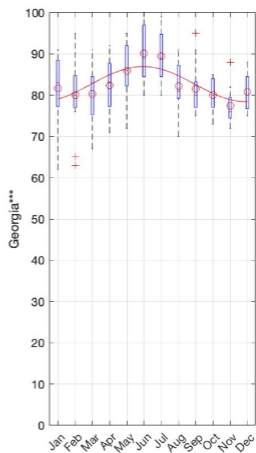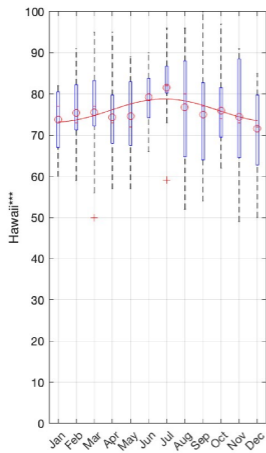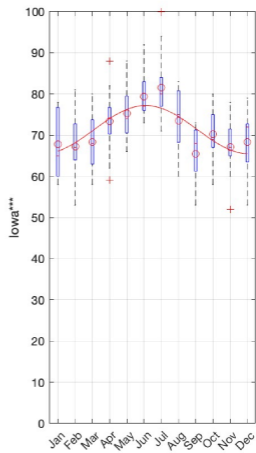

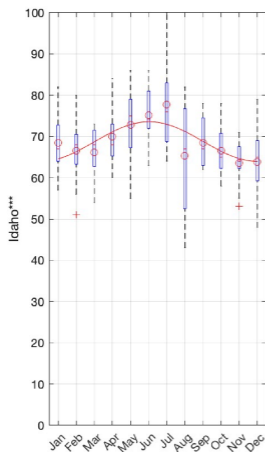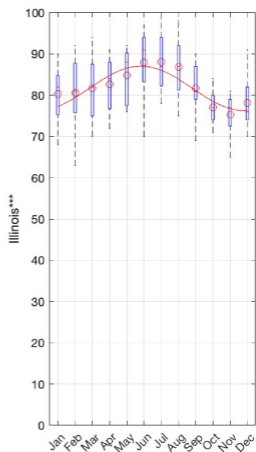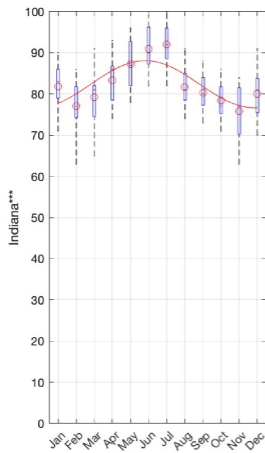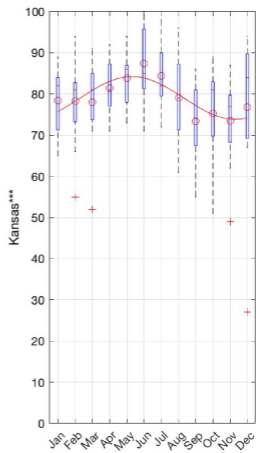

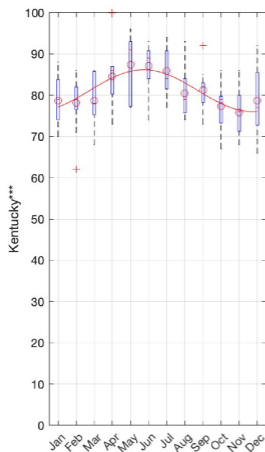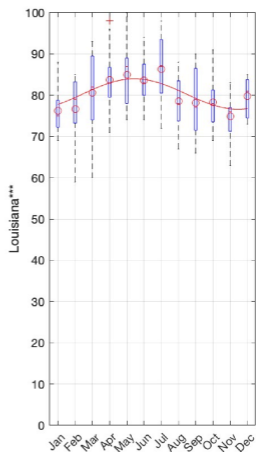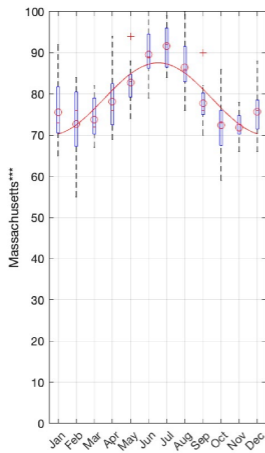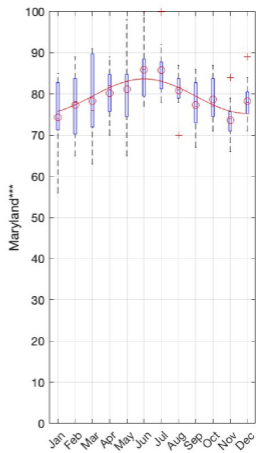

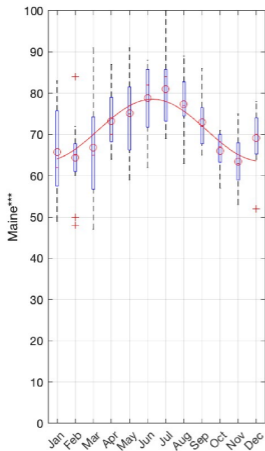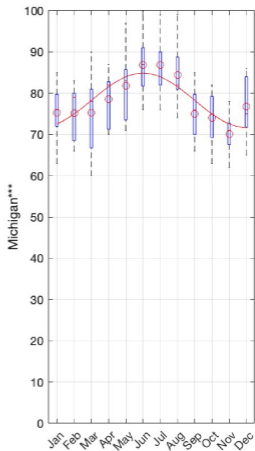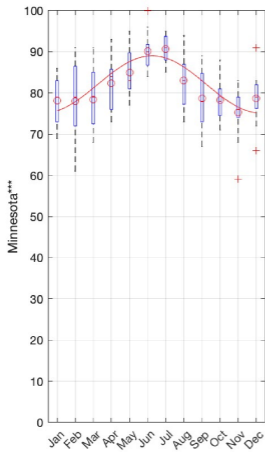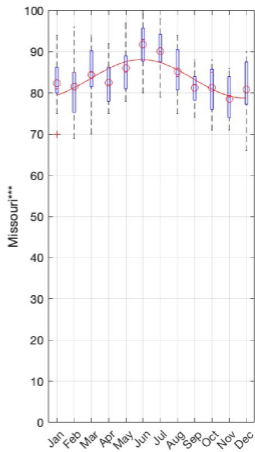

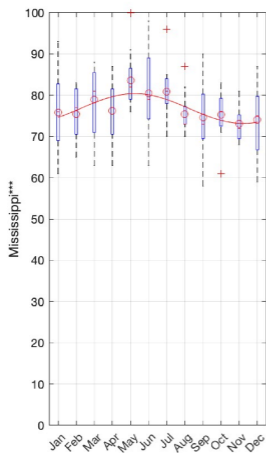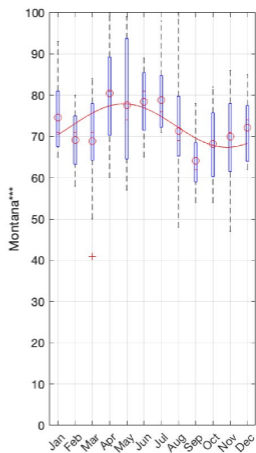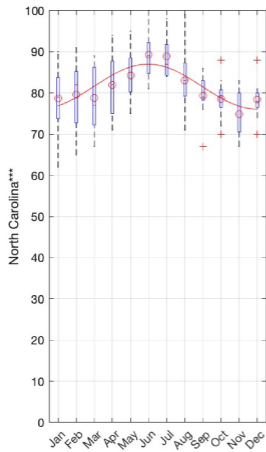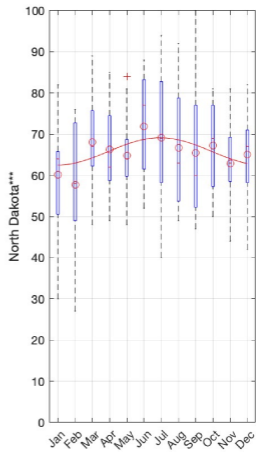

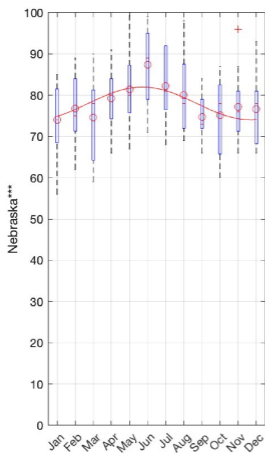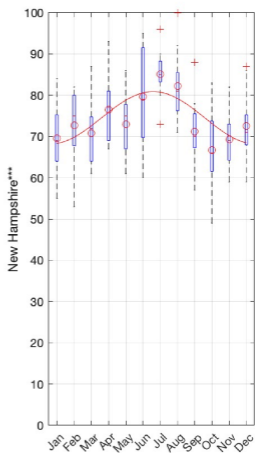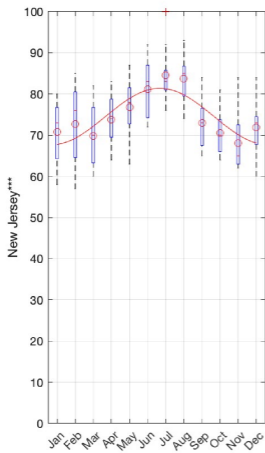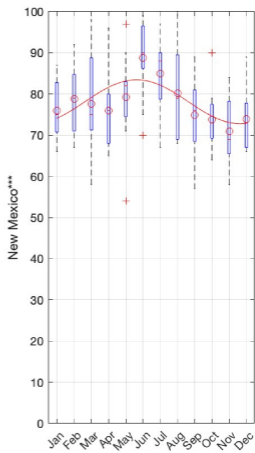

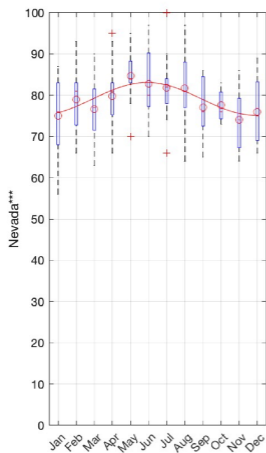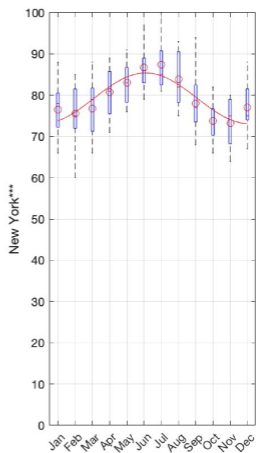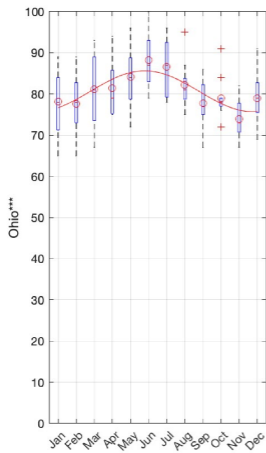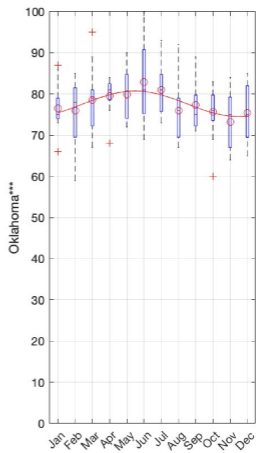

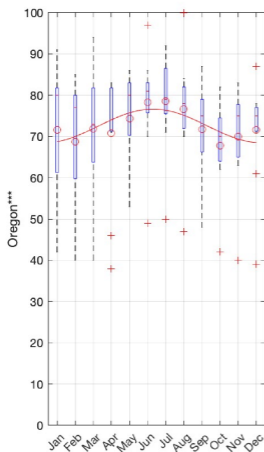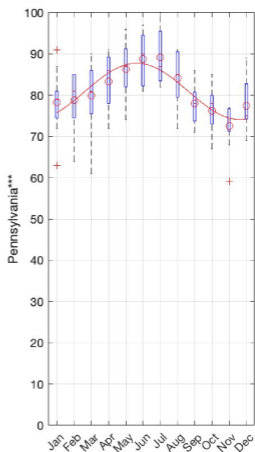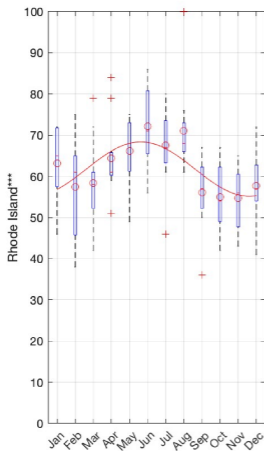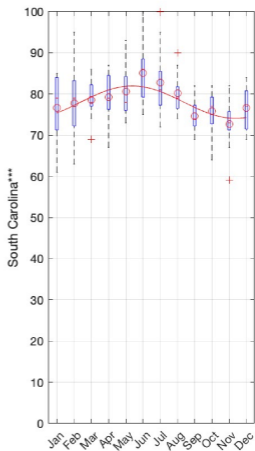

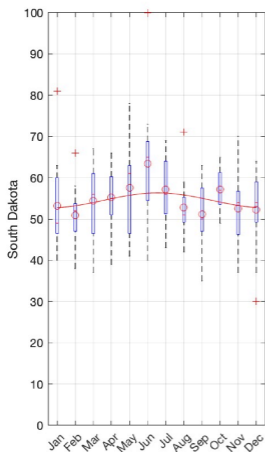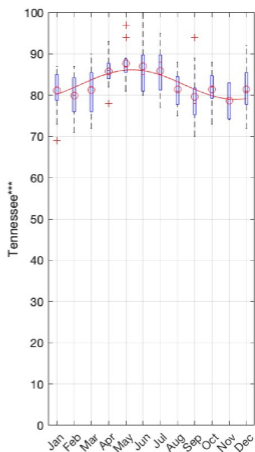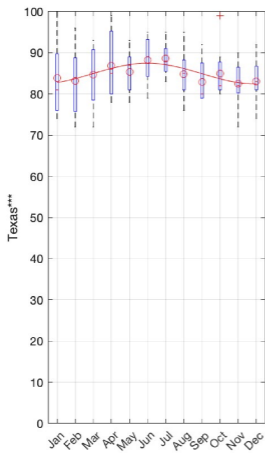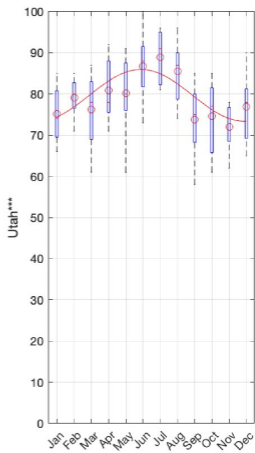

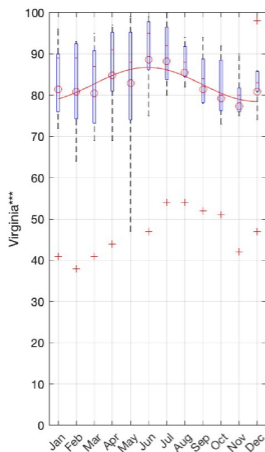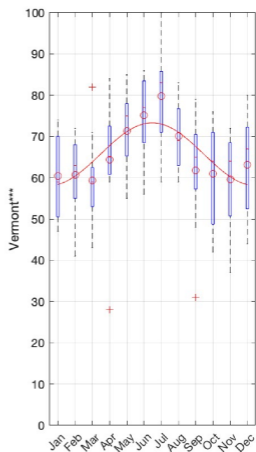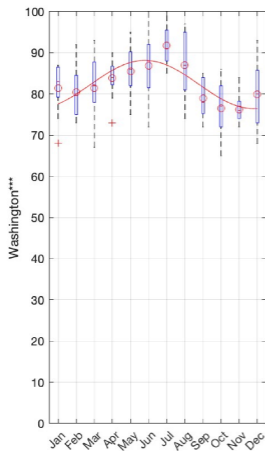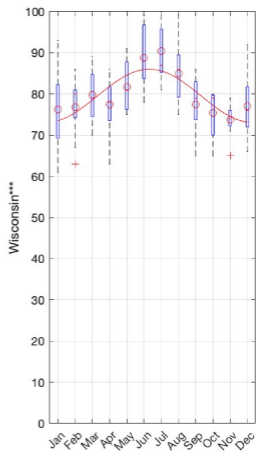

Supplement: Multimedia Appendix 3 [file jmir-v27-e75415-s003.pdf]

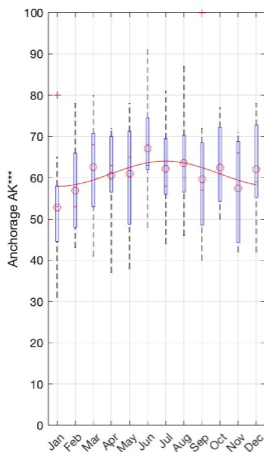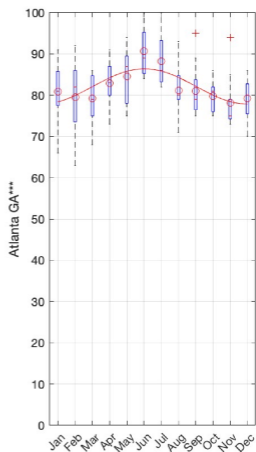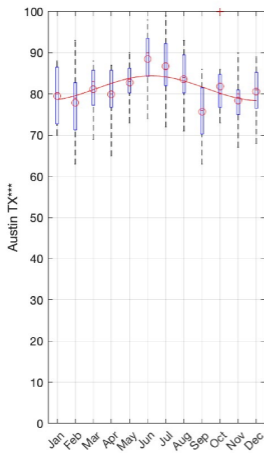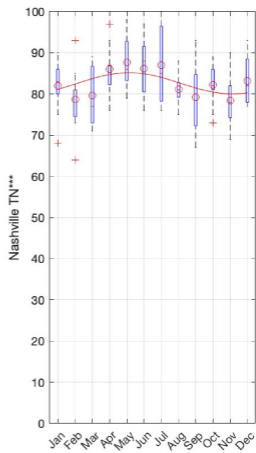

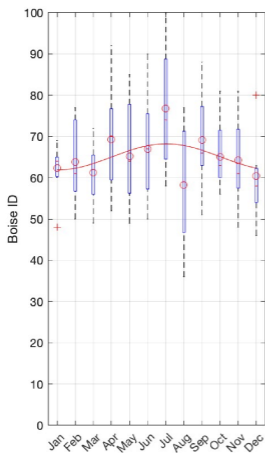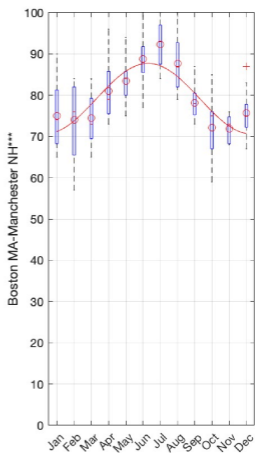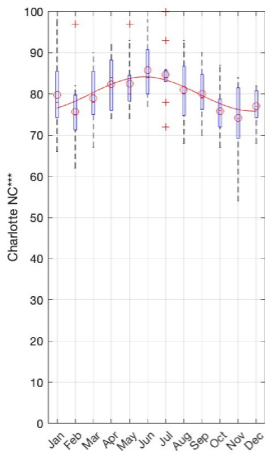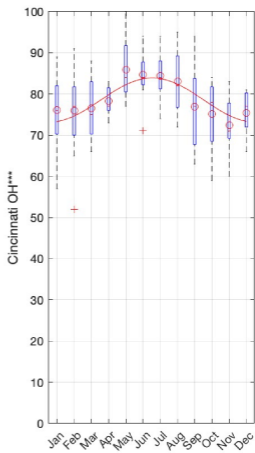

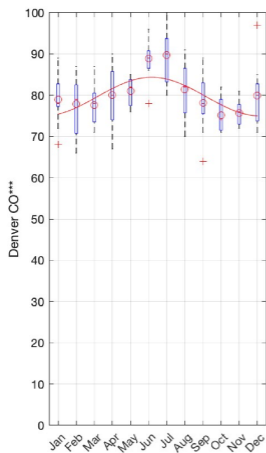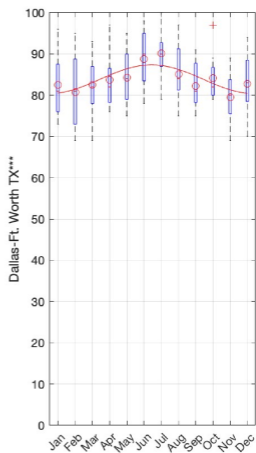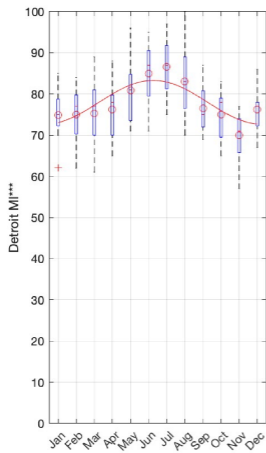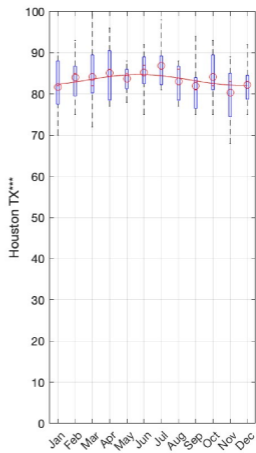

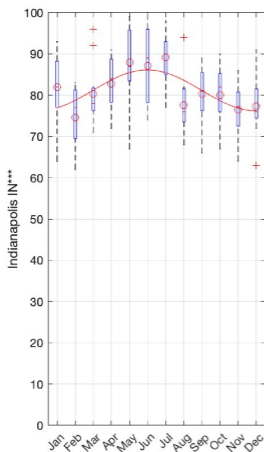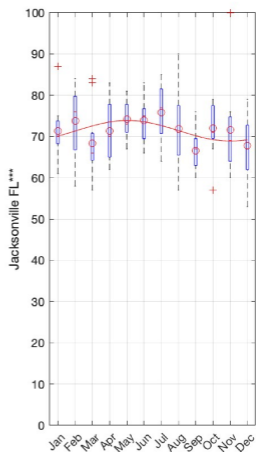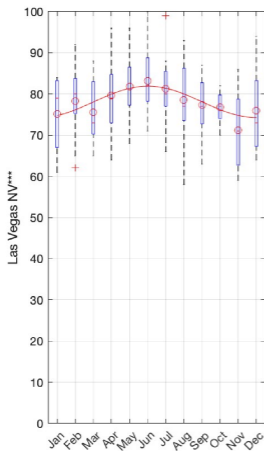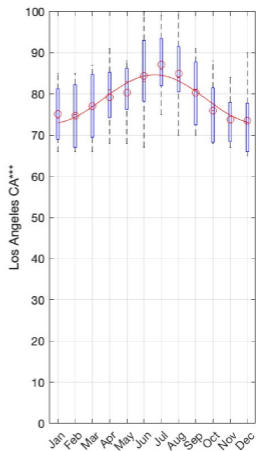

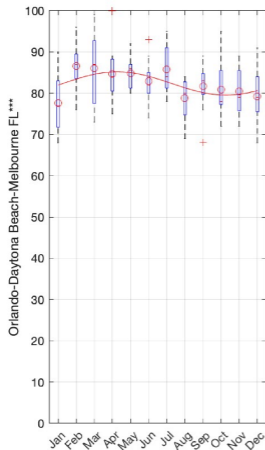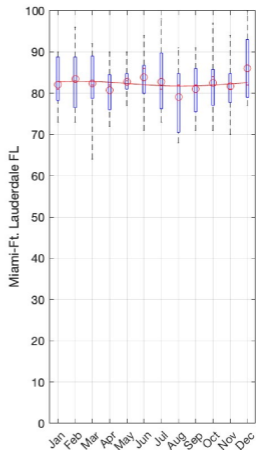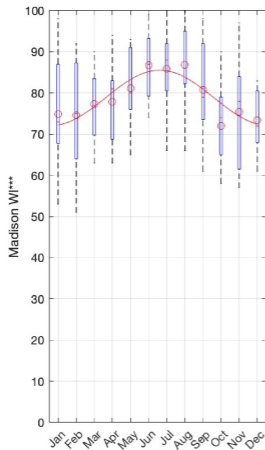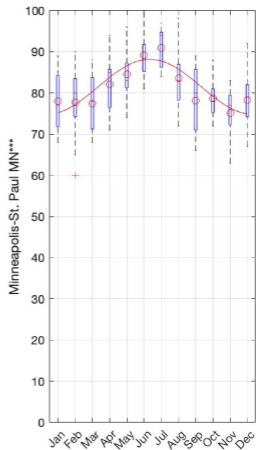

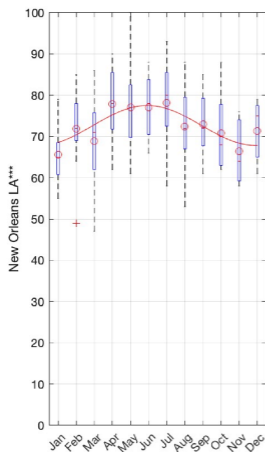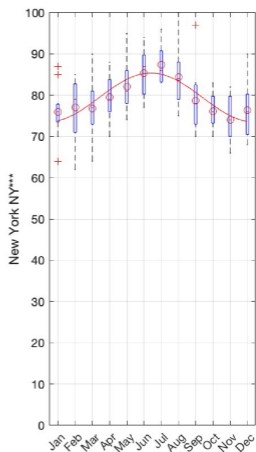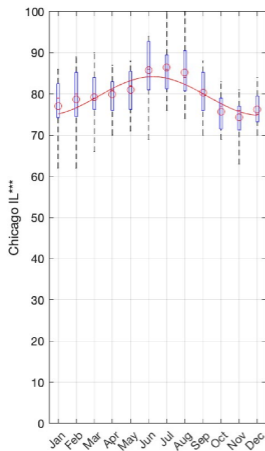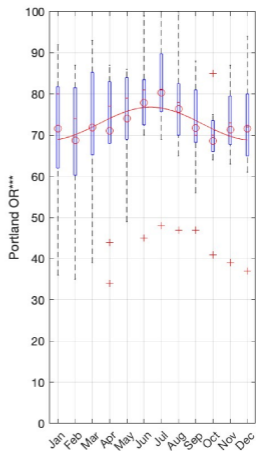

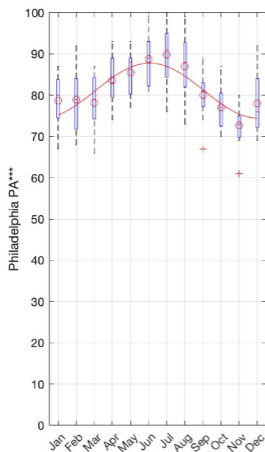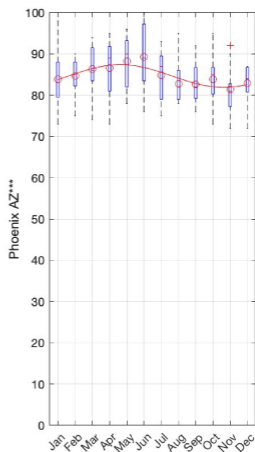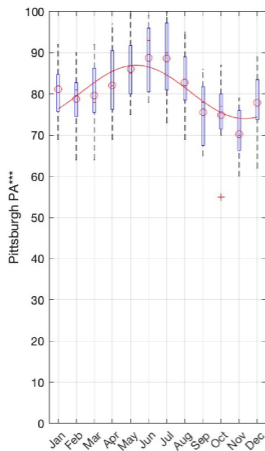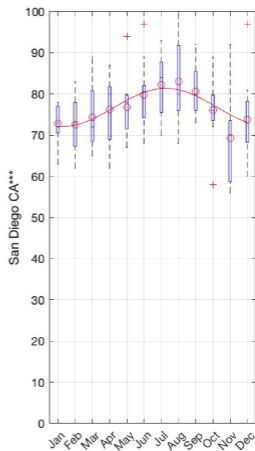

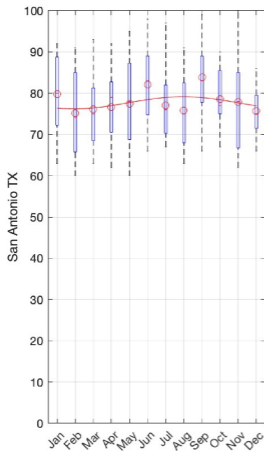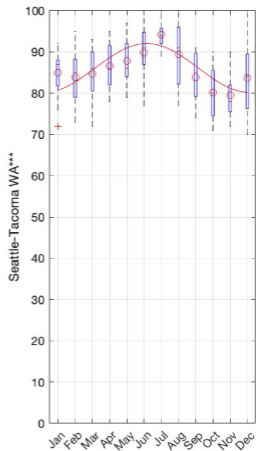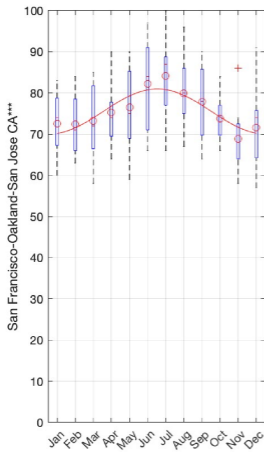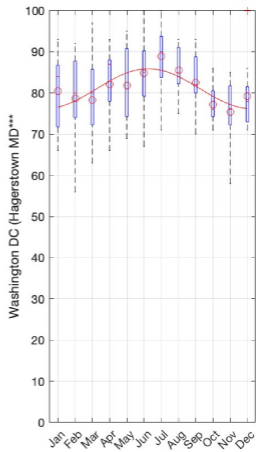

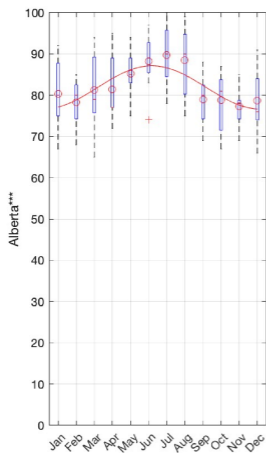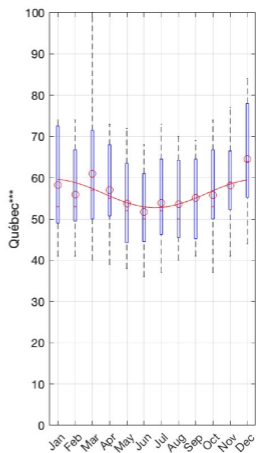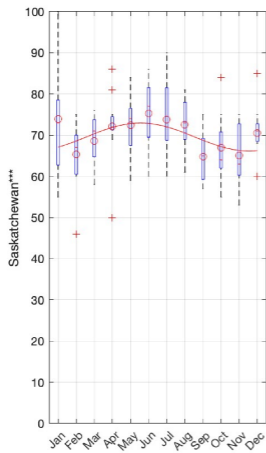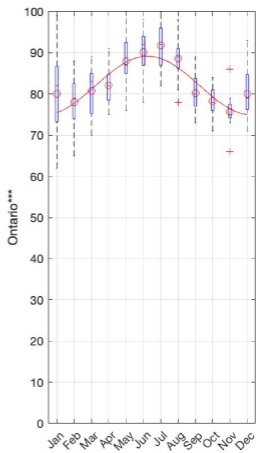

Supplement: Multimedia Appendix 4 [file jmir-v27-e75415-s004.pdf]

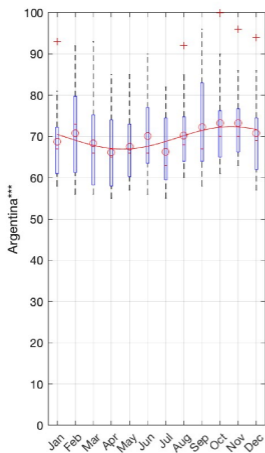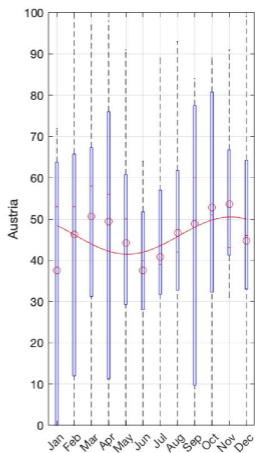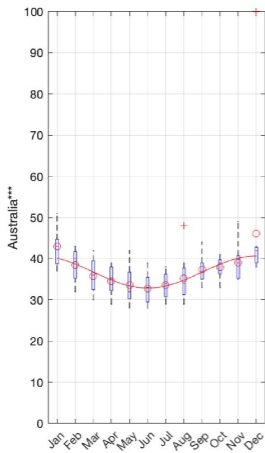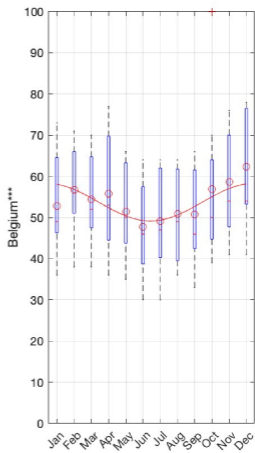

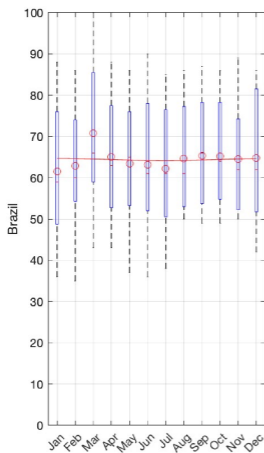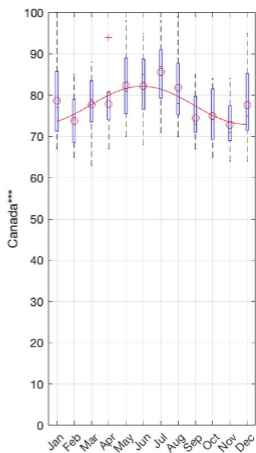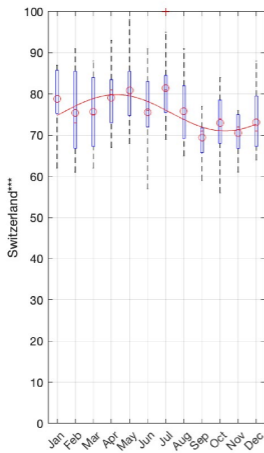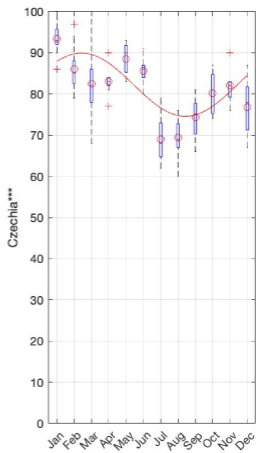

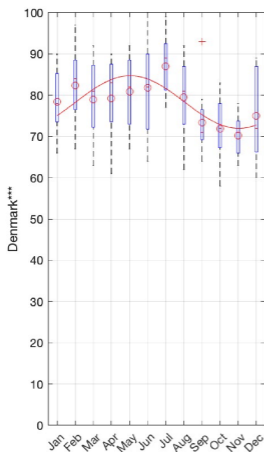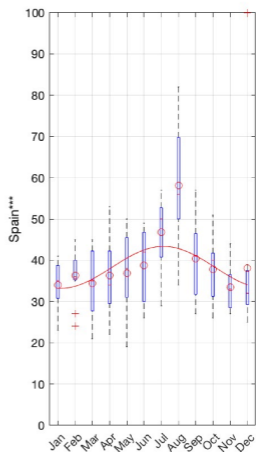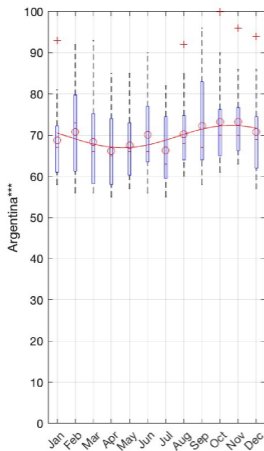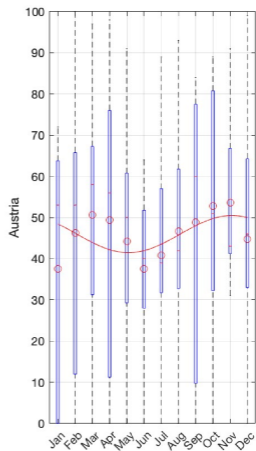

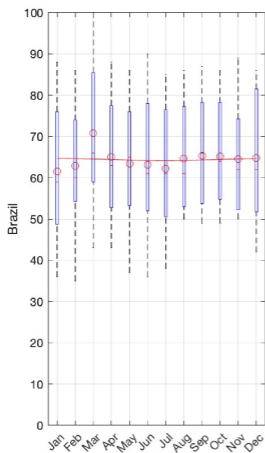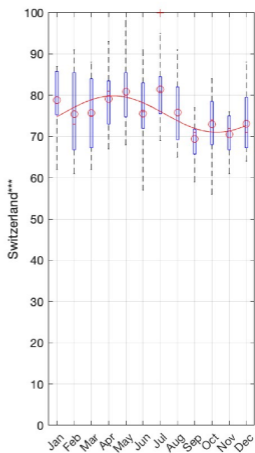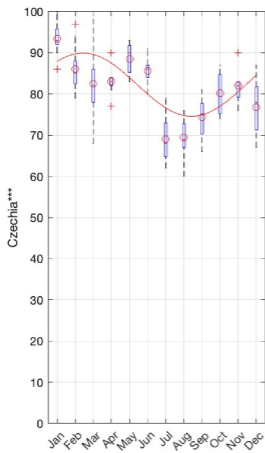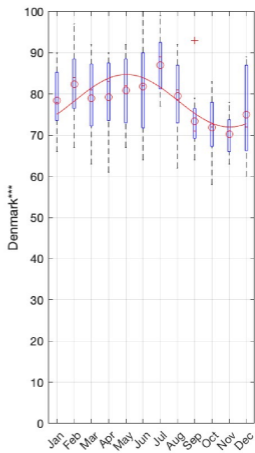

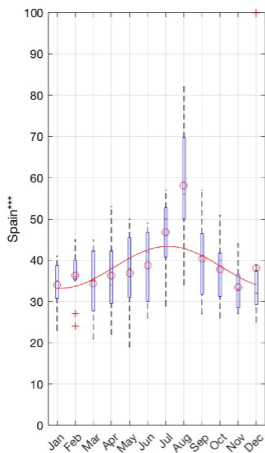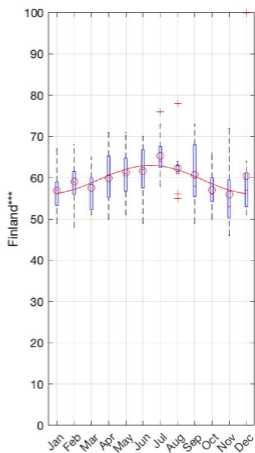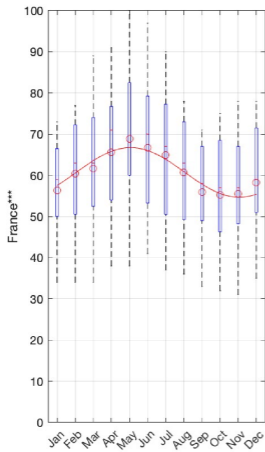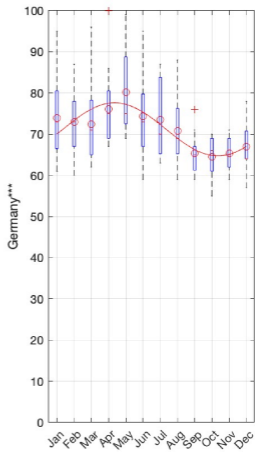

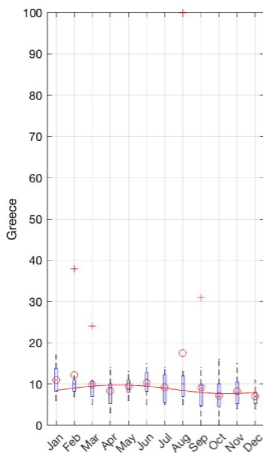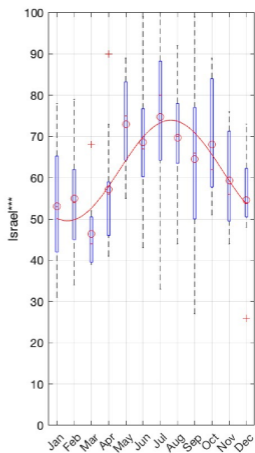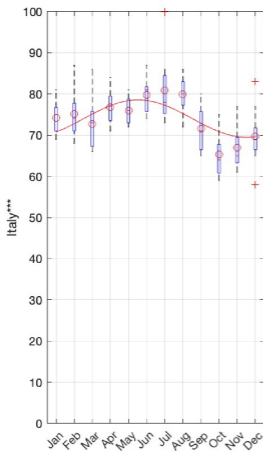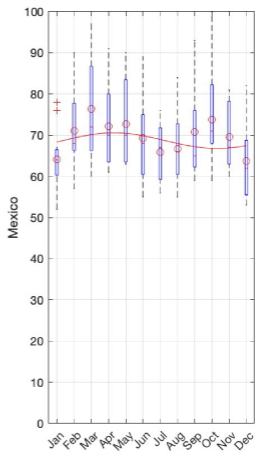

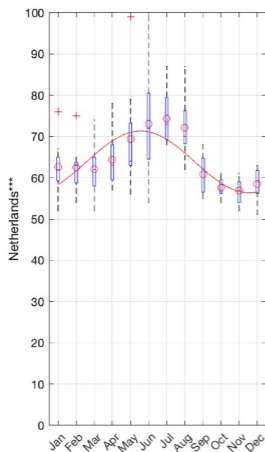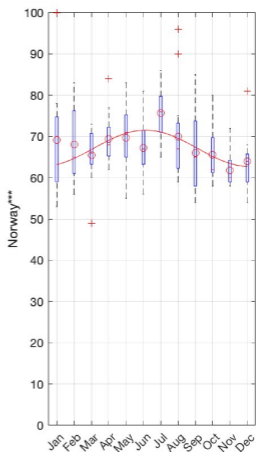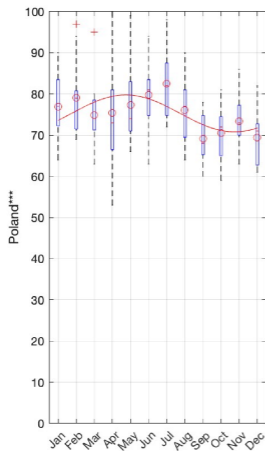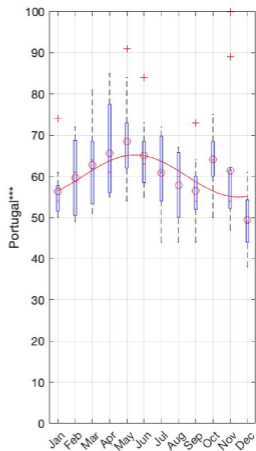

Supplement: Multimedia Appendix 5 [file jmir-v27-e75415-s005.pdf]

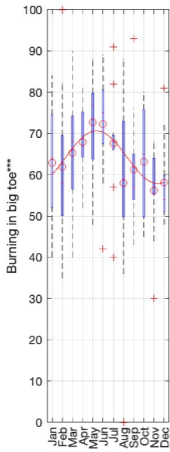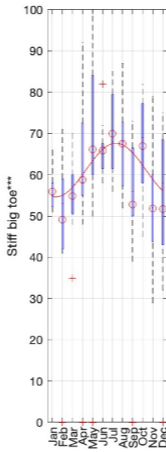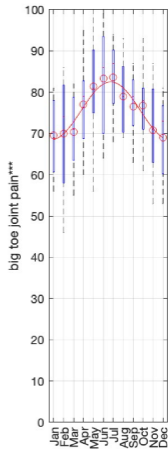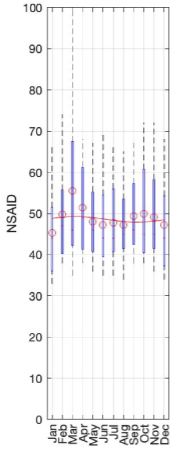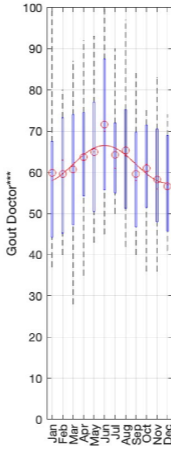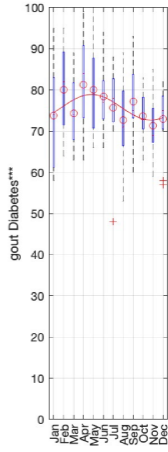

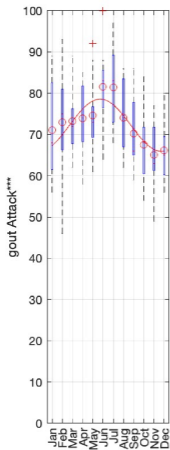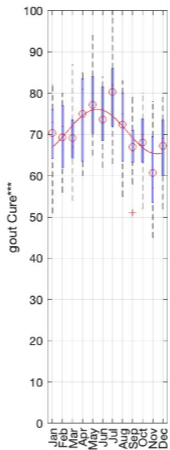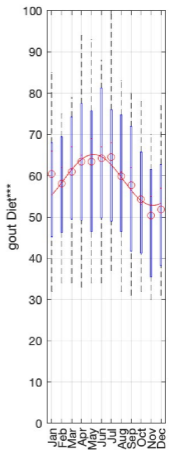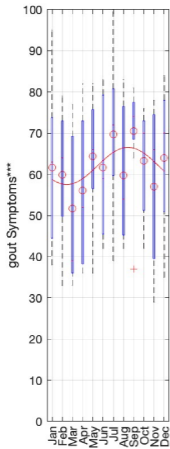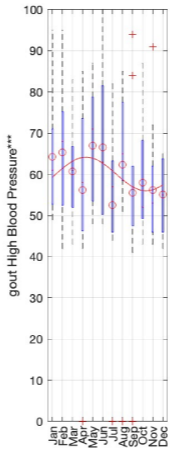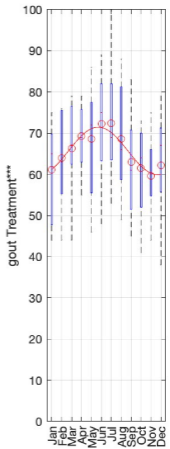

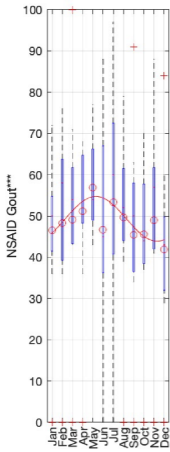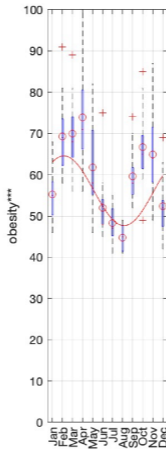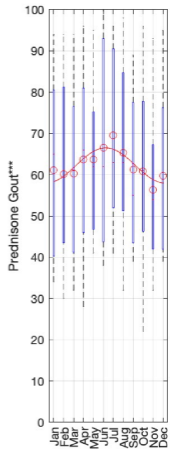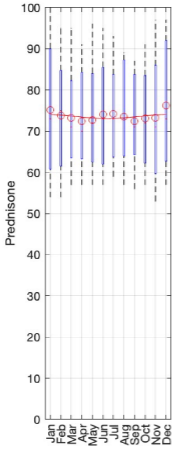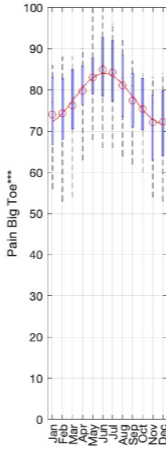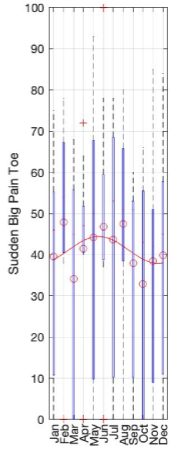

Supplement: Multimedia Appendix 6 [file jmir-v27-e75415-s006.pdf]
